# Mechanism of Mitophagy to Protect Yak Kidney from Hypoxia-Induced Fibrosis Damage by Regulating Ferroptosis Pathway

**DOI:** 10.3390/biom15040556

**Published:** 2025-04-09

**Authors:** Xuefeng Bai, Hongqin Lu, Rui Ma, Sijiu Yu, Shanshan Yang, Junfeng He, Yan Cui

**Affiliations:** 1 Department of Basic Veterinary Medicine, Faculty of Veterinary Medicine, Gansu Agricultural University, Lanzhou 730070, China; 107332001029@st.gsau.edu.cn (X.B.); luhongqin@xjnydx.wecom.work (H.L.); 107332001032@st.gsau.edu.cn (R.M.); yusj@gsau.edu.cn (S.Y.); 107332101038@st.gsau.edu.cn (S.Y.); hejf@gsau.edu.cn (J.H.); 2 Gansu Province Livestock Embryo Engineering Research Center, Department of Clinical Veterinary Medicine, Faculty of Veterinary Medicine, Gansu Agricultural University, Lanzhou 730070, China

**Keywords:** mitophagy, ferroptosis, renal fibrosis, hypoxia adaptation, yak

## Abstract

Renal fibrosis is a critical pathological feature of various chronic kidney diseases, with hypoxia being recognized as an important factor in inducing fibrosis. Yaks have long inhabited high-altitude hypoxic environments and do not exhibit fibrotic damage under chronic hypoxia. However, the underlying protective mechanisms remain unclear. This study compared the renal tissue structure and collagen volume between low-altitude cattle and high-altitude yaks, revealing that yaks possess a significantly higher number of renal tubules than cattle, though collagen volume showed no significant difference. Under hypoxic treatment, we observed that chronic hypoxia induced renal fibrosis in cattle, but did not show a significant effect in yaks, suggesting that the hypoxia adaptation mechanisms in yaks may have an anti-fibrotic effect. Further investigation demonstrated a significant upregulation of P-AMPK/AMPK, Parkin, PINK1, LC3Ⅱ/Ⅰ, and BECN1, alongside a downregulation of P-mTOR/mTOR in yak kidneys. Additionally, hypoxia-induced renal tubular epithelial cells (RTECs) showed increased expression of mitophagy-related proteins, mitochondrial membrane depolarization, and an increased number of lysosomes, indicating that hypoxia induces mitophagy. By regulating the mitophagy pathway through drugs, we found that under chronic hypoxia, activation of mitophagy upregulated E-cadherin protein expression while downregulating the expression of Vimentin, α-SMA, Collagen I, and Fibronectin. Simultaneously, there was an increase in SLC7A11, GPX4, and GSH levels, and a decrease in ROS, MDA, and Fe^2^⁺ accumulation. Inhibition of mitophagy produced opposite effects on protein expression and cellular markers. Further studies identified ferroptosis as a key mechanism promoting renal fibrosis. Moreover, in renal fibrosis models, mitophagy reduced the accumulation of ROS, MDA, and Fe^2^⁺, thereby alleviating ferroptosis-induced renal fibrosis. These findings suggest that chronic hypoxia protects yaks from hypoxia-induced renal fibrosis by activating mitophagy to inhibit the ferroptosis pathway.

## 1. Introduction

The kidneys are vital components of the urinary system, essential for maintaining homeostasis, regulating water-electrolyte and acid-base balance, and eliminating metabolic waste. Chronic kidney disease (CKD) is a prevalent condition that poses a significant threat to human health due to its high incidence and mortality rates. Kidney fibrosis is a hallmark of nearly all CKD and a major pathological factor driving progression to end-stage renal disease (ESRD) [1]. Research has shown that kidney fibrosis is closely associated with chronic inflammation, excessive extracellular matrix (ECM) deposition, fibroblast activation, hypoxia, and oxidative stress [2]. Among these factors, hypoxia plays a critical role in promoting renal fibrosis. Under hypoxic conditions, an insufficient oxygen supply activates hypoxia-inducible factors (HIFs), which regulate pathways such as TGF-β, Notch, PI3K/Akt, and NF-κB, inducing epithelial-to-mesenchymal transition (EMT) in RTECs, and thereby promoting fibrosis [3]. However, yaks, an economically important species that has long inhabited high-altitude hypoxic environments, have developed unique physiological mechanisms to adapt to low-oxygen environment. Previous studies have shown that under hypoxic conditions, the expression of HIF-1α is upregulated in yak RTEC [4]. Additionally, the expression level of HIF-1α in the lungs of yaks is significantly higher than that in cattle [5]. These results suggest that although yaks are exposed to hypoxic conditions, their unique hypoxia adaptation mechanisms enable them to maintain homeostasis. Therefore, exploring the mechanisms by which yak kidneys are protected from hypoxia-induced fibrotic damage will not only help to understand the physiological characteristics of high-altitude adaptive organisms, but also provide new insights into the pathological mechanisms of renal fibrosis.

Ferroptosis is a recently identified form of iron-dependent cell death. Studies have shown that the depletion of intracellular glutathione (GSH) leads to a reduced activity of glutathione peroxidase 4 (GPX4). As a result, lipid peroxides cannot be metabolized through GPX4-catalyzed reduction reactions, and Fe^2^⁺ oxidizes lipids in a Fenton-like manner, generating large amounts of reactive oxygen species (ROS), thereby promoting ferroptosis [6,7]. Therefore, the accumulation of Fe^2^⁺ and lipid peroxidation are key characteristics of ferroptosis [8]. Ferroptosis plays a critical role in the progression of various systemic diseases, such as myocardial ischemia/reperfusion injury (IRI), liver fibrosis, type 1 diabetes, acute kidney injury (AKI), and renal cell carcinoma [9]. Studies has shown that characteristics of ferroptosis, including excessive iron accumulation and lipid peroxidation, are present during the development of kidney diseases [10]. Animal experiments have demonstrated that the use of ferroptosis inhibitors (Ferrostatin-1) can attenuate the progression of fibrosis and suppress the release of pro-fibrotic factors in a mouse renal fibrosis model (UUO) [11,12]. Furthermore, in a CKD rat model, induced by 5/6 nephrectomy, the activation or inhibition of ferroptosis resulted in an increase or decrease, respectively, in renal fibrosis markers [13]. These findings suggest that inhibiting ferroptosis is a potential therapeutic target for treating renal fibrosis.

Mitochondria, as the energy factories of cells, play a crucial role in cellular function. When mitochondrial function is impaired, it can lead to the accumulation of ROS, which in turn triggers a series of pathological reactions [14]. To maintain mitochondrial quality and cellular health, cells activate the mitophagy pathway to selectively remove damaged or dysfunctional mitochondria. Notably, our previous research also demonstrated that mitophagy can regulate metabolic pathways to inhibit apoptosis in yak RTECs and maintain cellular homeostasis [15]. Furthermore, with the advancement of research into the mechanisms of mitophagy, it has been shown to hold potential in the prevention and treatment of various diseases, especially those associated with oxidative stress, cell death, and fibrosis [16,17]. Studies have shown that Farrerol can alleviate cisplatin-induced CKD by activating the Parkin-mediated mitophagy pathway [18]. The accumulation of ROS is one of the primary causes of ferroptosis and renal fibrosis, but mitophagy can reduce ROS production by degrading damaged mitochondria. It has been reported that AKI in RTECs induced by cisplatin leads to ferroptosis, while activation of mitophagy can alleviate ferroptosis and kidney injury [19].

However, the regulatory mechanisms of mitophagy in protecting yak kidneys from fibrotic damage, particularly its role in modulating the ferroptosis pathway, have not yet been reported. Therefore, this study aims to elucidate the regulatory mechanisms of mitophagy in yak renal fibrosis and its effects on the ferroptosis pathway. The findings are expected to offer new theoretical insights and potential targets for understanding high-altitude adaptation mechanisms and for the prevention and treatment of hypoxia-related diseases.

## 2. Materials and Methods

### 2.1. Animals and Ethics

In this study, adult male yaks (4 years old, *n* = 6) from high-altitude regions (altitude > 3000 m) and adult male cattle (4 years old, *n* = 6) from low-altitude areas (1000 m) were selected as animal samples. All animals were clinically healthy. Tissue samples designated for histological analysis were fixed in 4% paraformaldehyde (PFA) at room temperature for one week. Samples intended for Western blot analysis were stored at −80 °C. For cell culture experiments, fresh kidney tissues were immediately immersed in sterile physiological saline and transported to the laboratory for primary cell culture. All experimental procedures were handled according to the Animal Ethics Procedures and Guidelines of the People’s Republic of China. This study was also approved by the Animal Ethics Committee of Gansu Agricultural University (GSAU-Eth-VMC-2023-004).

### 2.2. Histological Staining

Kidney samples from adult yaks and yellow-cattle were fixed in 4% PFA for one week. Subsequently, the samples were cut into 1 cm^3^ pieces, dehydrated for 24 h, embedded in paraffin, and sectioned into 4 μm-thick paraffin sections. Deparaffinization of these sections was achieved using xylene and alcohol in preparation for histological staining. H&E, Masson and Sirius red staining were performed following the instructions provided by the relevant reagent manufacturer (Solarbio, Beijing, China). We selected at least five slices from each group of samples. For every slice, three fields of view were randomly chosen, and photographs were taken using an Olympus-DP73 optical microscope (Olympus, Tokyo, Japan). To quantify collagen volume after Masson’s trichrome or Sirius red staining, we utilized Image-J 1.54software (National Institutes of Health, Bethesda, MD, USA) to analyze the percentage of the stained area in each randomly selected field of view relative to the total area. Importantly, all sample analyses were conducted in a blinded manner.

### 2.3. Immunohistochemistry Staining of Kidney Tissues

Paraffin-embedded tissue sections (4 μm) were deparaffinized using xylene and rehydrated through a series of graded ethanol solutions. After washing with distilled water, antigen retrieval was performed by boiling the sections in citrate buffer (pH 6.0) for 15 min. To block endogenous peroxidase activity, the sections were incubated with 3% hydrogen peroxide at room temperature for 10 min. Subsequently, the sections were incubated at 37 °C for 2 h with anti-P-AMPK (1: 300, AF3423, Affinity, Shanghai, China), anti-P-mTOR (1:300, AF3308, Affinity), anti-Parkin (1:300, 14060-1-AP, Proteintech, Wuhan, China), anti-PINK1 (1:100, DF7742, Affinity), anti-LC3 (1:300, 14600-1-AP, Proteintech), and anti-BECN1 (1:25, AF5128, Affinity) antibodies. Following the PBS washes, the slides were treated with a biotinylated secondary antibody and incubated at 37 °C for 15 min, and finally 3,3-diaminobenzidine (DAB) (Bios, Beijing, China) was used for color development. The sections were counterstained with hematoxylin. For negative controls, PBS (pH 7.4) was substituted for the primary antibody, while all other conditions remained unchanged. Photographs of each section were captured using an Olympus DP73 optical microscope (Olympus, Tokyo, Japan).

### 2.4. Cell Culture and Hypoxia Induction

Yak and cattle RTECs were primary cells, and the culturing methods have been detailed in our previous studies [15]. RTECs were cultured in DMEM/F12 medium (Gibco, Grand Island, NE, USA) and contained 10% fetal bovine serum (FBS, Gibco, USA), penicillin (100 IU/mL, Solarbio, Beijing, China), and streptomycin (100 µg/mL, Solarbio, Beijing, China). The cells were cultured in an incubator at 37 °C and 5% CO_2_. RTECs were exposed to normoxic conditions (37 °C, 21% O_2_, 5% CO_2_) and hypoxic conditions (37 °C, 5% O_2_, 5% CO_2_), respectively. Samples were collected every 24 h from the hypoxic treatment group for a total of 72 h to analyze changes at different hypoxic time points.

### 2.5. Western Blotting

After washing the kidney tissue samples and the RTEC samples with PBS, radioimmunoprecipitation assay lysis buffer (Solarbio, Beijing, China) was added to lyse the samples. The extracted protein was mixed with protein loading buffer (Solarbio, Beijing, China) and heated in a metal bath at 100 °C for 10 min to denature the proteins. Subsequent experiments were performed according to the Western blot protocol. Briefly, equal amounts of protein samples were separated by SDS-PAGE, followed by transfer to a 0.22 μm/0.45 μm PVDF membrane (Millipore, Burlington, MA, USA). The membrane was blocked with Tris-buffered saline with 0.1% Tween-20 and 5% BSA at room temperature for 2 h. Subsequently, the membrane was incubated overnight at 4 °C with specific primary antibodies: anti-P-AMPK (1:1000, AF3423, Affinity, Shanghai, China), anti-AMPK (1:1000, AF3423, Affinity), anti-P-mTOR (1:800, AF3308, Affinity), anti-mTOR (1:800, AF6308, Affinity), anti-LC3 (1:2000, 14600-1-AP, Proteintech, Wuhan, China), anti-Parkin (1:1500, 14060-1-AP, Proteintech), anti-PINK1 (1:1000, DF7742, Affinity), anti-E-cadherin (1:2000, AF0131, Affinity), anti-Fibronectin(1:2500, 15613-1-AP, Proteintech), anti-α-SMA(1:2000, AF0048, Beyotime, Shanghai, China), anti-Collagen Ⅰ (1:1500, AF7001, Affinity), anti-GPX4 (1:2000, 67763-1-Ig, Proteintech), anti-SLC7A11(1:1000, 26864-1-AP, Proteintech), and anti-β-actin (1:3000, bs-0061R, Bioss, Beijing, China). After washing the PVDF membrane with PBST, incubate it with HRP-conjugated secondary antibodies (goat anti-rabbit IgG or goat anti-mouse IgG, 1:3000, Bioss) at 37 °C for 1 h. The bands were then visualized using an enhanced chemiluminescent (ECL) detection reagent (Vazyme, Nanjing, China) and imaged with the Amersham Imager 600 chemiluminescence imaging system (GE Healthcare BioSciences AB, Uppsala, Sweden). The images were quantified by densitometry using Image J software. The relative expressions of the proteins were normalized to β-actin.

### 2.6. Mitochondrial-Lysosomal Co-Localization

For mitochondrial and lysosomal localization in RTECs, the following procedure was used: cells were seeded in 6-well plates and incubated with Mito-Tracker Green (Beyotime, Shanghai, Beijing) at 37 °C for 30 min to stain the mitochondria. After washing, Lyso-Tracker Red (Beyotime, Shanghai, Beijing) was added and incubated at 37 °C for an additional 15 min to label the lysosomes. Nuclei were stained with Hoechst 33342 (Beyotime, Shanghai, Beijing). Images were captured using a fluorescence microscope (Olympus-DP71, Tokyo, Japan). This protocol allows for simultaneous visualization of mitochondria and lysosomes.

### 2.7. Drug Treatment

In the drug treatment experiments, RTECs were seeded into 96-well plates. After hypoxia induction for 48 h, cells were treated with specific drugs at varying concentrations to evaluate their effects under hypoxic conditions. The drugs included Mdivi-1 (mitophagy inhibitor, Med Chem Express): 0, 25, 50, 100, 200, 400, and 800 µM; Metformin (mitophagy activator, Med Chem Express): 0, 1, 2, 4, 8, 16, and 32 mM; RSL3 (ferroptosis activator, Med Chem Express): 0, 0.5, 1, 2, 4, and 8 µM; Ferrostatin-1 (ferroptosis inhibitor, Med Chem Express): 0, 5, 10, 20, 40, and 100 µM; and Cisplatin (renal fibrosis activator, Med Chem Express): 0, 5, 10, 20, 40, 80, and 100 µM. Following the drug treatment, cells were maintained under hypoxic conditions for an additional 24 h. The CCK-8 assay was used to assess cell viability, and the results were analyzed to determine the optimal concentrations of each drug for subsequent experiments.

### 2.8. Mitochondrial Membrane Potential Detection

RTECs were seeded into 6-well plates and subjected to hypoxic conditions or drug treatments according to the experimental design. Following treatment, cells were collected by trypsin digestion and resuspended in PBS. A 100 µL aliquot of the cell suspension was transferred into a flow cytometry tube, and 300 µL of prepared JC-10 probe solution (AAT Bioquest, Pleasanton, CA, USA) was added. The samples were incubated in the dark for 30 min. After incubation, unbound probes were washed off, and the mitochondrial membrane potential was assessed using a flow cytometer (Beckman, Brea, CA, USA). The resulting data were analyzed with Modfit LT 5.0 software.

### 2.9. ROS Assessment

To use the DCFH-DA probe (Solarbio, Beijing, China) to detect ROS in RTECs, dilute the probe to a final concentration of 1 μmol/L in serum-free culture medium. After removing the cell culture medium, add 1 mL of the diluted DCFH-DA solution to the cells and incubate at 37 °C in a cell culture incubator for 30 min. After incubation, wash cells thoroughly with PBS to remove unincorporated DCFH-DA probe. ROS staining results can be observed using a fluorescence microscope (Olympus DP 71, Tokyo, Japan) or ROS levels can be assessed using a flow cytometer (Beckman, Brea, CA, USA).

### 2.10. Lipid Peroxidation Staining

RTECs were seeded in a 6-well plate and cultured for 24 h before drug treatment. After treatment, an appropriate amount of Lipid Peroxidation Probe (BODIPY 581/591 C11, Beyotime, Shanghai, China) was added to each well at a final concentration of 2 μM. The cells were then incubated in a cell culture incubator for 20 min. After incubation, the supernatant was removed, and the cells were washed twice with PBS. Subsequently, 2 mL of PBS was added, and fluorescence imaging was performed using a fluorescence microscope. The reduced form of BODIPY 581/591 C11 emits red fluorescence, with maximum excitation/emission wavelengths of 581/591 nm. Upon oxidation, the fluorescence shifts to green, with an excitation/emission peak at 488/510 nm.

### 2.11. Determination of GSH, MDA and Fe^2+^

The expression levels of GSH, MDA, and Fe^2^⁺ are important indicators for evaluating ferroptosis. In this study, the Reduced Glutathione (GSH) Content Assay Kit, Malondialdehyde (MDA) Content Assay Kit, and Ferrous Ion (Fe^2^⁺) Content Assay Kit (Solarbio, Beijing, China) were used to detect the levels of GSH, MDA, and Fe^2^⁺ in RTECs after drug treatment. The detection was carried out according to the reagent manufacturer’s instructions. Each experiment was performed in triplicate to ensure the reliability and reproducibility of the results.

### 2.12. Immunofluorescence

RTECs were seeded onto coverslips and fixed with 4% PFA for 30 min. To increase cell membrane permeability, cells were incubated with 0.5% Triton X-100 (Beyotime, Shanghai, China) at room temperature for 10 min. Afterward, the cells were blocked with 5% BSA for 1 h to prevent nonspecific binding. Next, primary antibodies against E-cadherin (1:250, AF0131, Affinity), β-catenin (1:200, AF6266, Affinity) and Vimentin (1:500, ab8069, Abcam, Cambridge, MA, USA) were added and incubated overnight at 4 °C. After the incubation, secondary antibodies, anti-Rabbit IgG (H+L)-Alexa Fluor 594 (A-11012, Thermo, Waltham, MA, USA) and anti-Mouse IgG (H+L)-Alexa Fluor 488 (A-11001, Thermo, Waltham, MA, USA), were applied for 1 h at room temperature in the dark. Finally, cell nuclei were stained with DAPI (10 μg/mL). Images were captured using a fluorescence microscope (Olympus DP71, Tokyo, Japan).

### 2.13. Fluorescence Staining of Autophagosomes

Ad-mCherry-GFP-LC3B was used to detect autophagosome formation in yak RTECs. Briefly, RTECs were seeded in 6-well plates. When the cell confluence reached 70–80%, the cells were infected with Ad-mCherry-GFP-LC3B adenovirus (Beyotime, Shanghai, China). After treatment, the nuclei were stained with DAPI (10 µg/mL) for 5 min. The intracellular fluorescence of Ad-mCherry-GFP-LC3B was observed under a fluorescence microscope (Olympus DP 71, Tokyo, Japan).

### 2.14. Statistics Analyses

The data are presented as the means ± standard errors of the means (S.E.M.) from three independent experiments and were analyzed using GraphPad Prism 8.3.0 software (GraphPad Software, San Diego, CA, USA). Differences between two groups were analyzed using two-tailed Student’s *t* tests. For comparisons involving multiple groups with more than one variable, two-way ANOVA followed by post hoc Tukey’s test was applied. A *p* value < 0.05 was considered to indicate statistical significance.

## 3. Results

### 3.1. Effects of High-Altitude Hypoxic Environment on Renal Fibrosis and Mitophagy in Yaks

**Figure 1:**
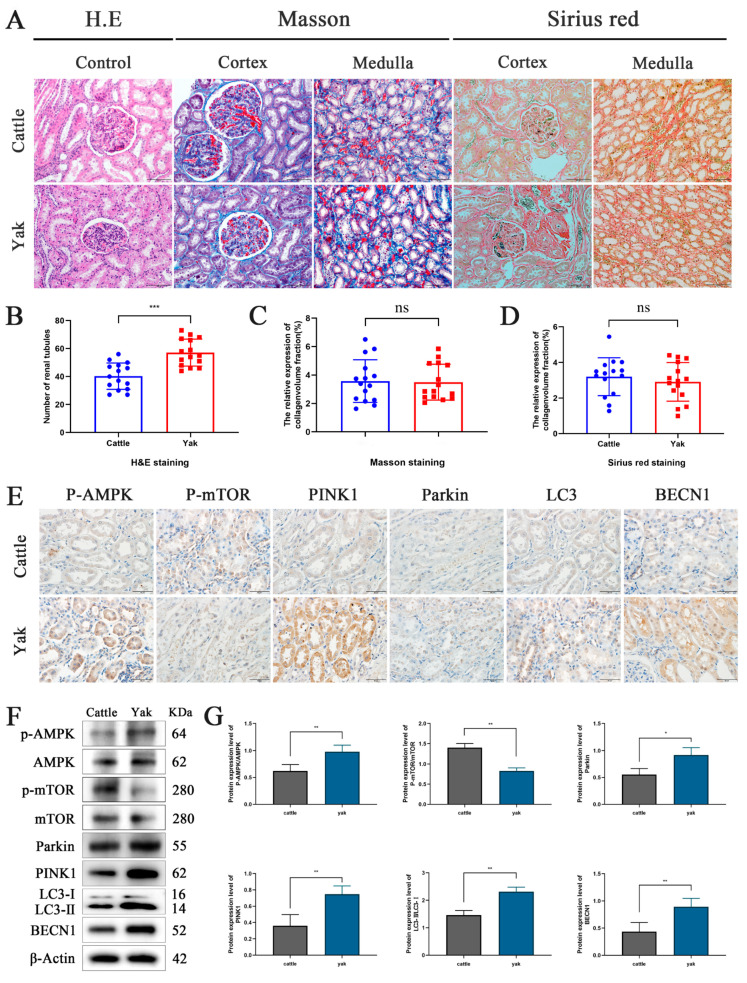
Histological staining and analysis of the effect of hypoxia on mitophagy-related proteins. (**A**) Hematoxylin and Eosin (H&E) staining showed the nucleus in blue-purple and the cytoplasm in pink. Masson staining highlighted collagen fibers in light blue, cytoplasm, and red blood cells in red, with blue-purple nuclei. Sirius Red staining revealed brownish-brown nuclei and red collagen fibers. Magnification: ×200; Scale = 100 μm. (**B**–**D**) Analysis of the number of renal tubules and collagen volume in cattle and yaks. (**E**) Localization and distribution of P-AMPK, P-mTOR, Parkin, PINK1, LC3 and BECN1 proteins in cattle and yak kidneys. Magnification: ×40, Bar = 50 µm. (**F**,**G**) Western blot analysis was used to assess the expression levels of P-AMPK/AMPK, P-mTOR/mTOR, Parkin, PINK1, LC3 Ⅱ/Ⅰ and BECN1 proteins in the kidney. The original WB images are shown in Figure S1. All data are presented as means ± SEM, ns = not significant, * *p* < 0.05; ** *p* < 0.01; *** *p* < 0.001.

To investigate the impact of high-altitude hypoxic environment on the kidney tissue structure and fibrosis development, we conducted a comparative analysis of kidney structure and collagen volume between yaks and cattle. H&E staining results demonstrated that the kidney tissue architecture of cattle and yaks is essentially similar (Figure 1A). However, the number of renal tubules in yak kidneys is significantly higher than that in cattle, indicating that yak kidneys possess superior reabsorption capacity and enhanced self-regulatory ability (Figure 1B). Masson and Sirius red staining indicated varying degrees of fibrosis in the kidney cortex and medulla of both yaks and cattle (Figure 1A). However, the data showed that there was no significant difference in renal fibrosis between yaks and cattle (Figure 1C,D). Mitophagy is an important mechanism for inhibiting renal fibrosis. Immunohistochemical staining results indicate that P-AMPK, P-mTOR, Parkin1, PINK1, LC3, and BECN1 are significantly expressed in renal tubular epithelial cells (Figure 1E). By comparing the expression levels of mitophagy-related proteins in the kidneys of yaks and cattle, the results showed that compared with cattle, the expression levels of P-AMPK/AMPK, Parkin1, PINK1, LC3Ⅱ/Ⅰ and BECN1 in the kidneys of yaks were significantly increased, while the expression level of P-mTOR/mTOR was significantly decreased (Figure 1F,G). These results indicate that the high-altitude hypoxic environment induces mitophagy in yak kidneys. Based on these findings, we hypothesize that the high-altitude hypoxic environment protects yak kidneys from hypoxia-induced fibrosis by inducing mitophagy.

### 3.2. Regulatory Effects of Hypoxia on Renal Fibrosis in Cattle and Yaks

**Figure 2:**
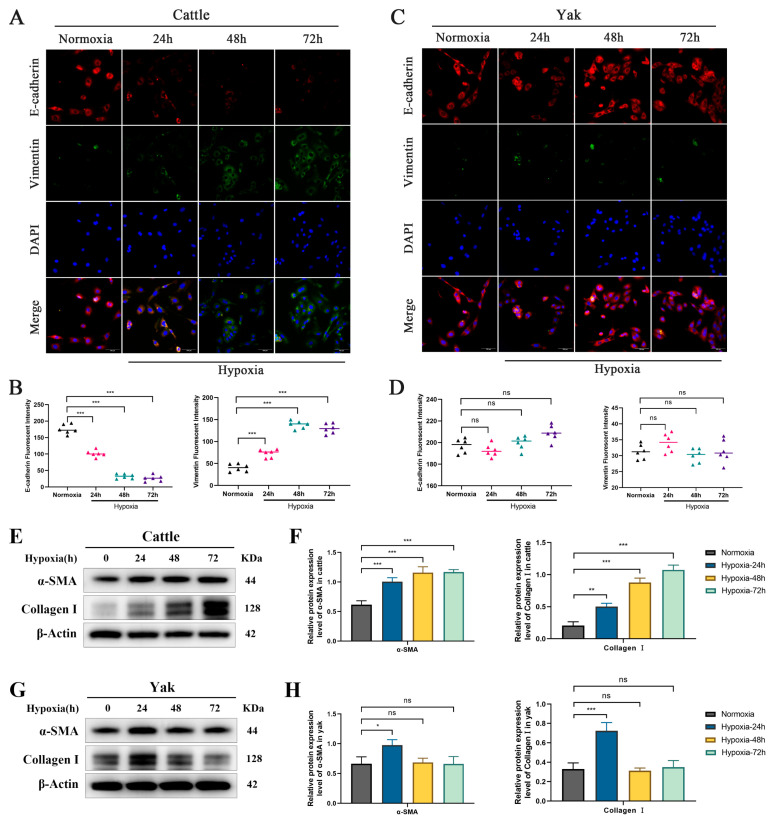
Response of cattle and yaks to hypoxia. (**A**) Immunofluorescence detection of E-cadherin and Vimentin in cattle RTECs under hypoxia at 0 h, 24 h, 48 h, and 72 h. Magnification: ×200, scale bar = 100 μm. (**B**) Relative expression analysis of E-cadherin and Vimentin in cattle RTECs. (**C**) Immunofluorescence detection of E-cadherin and Vimentin in yak RTECs under hypoxia at 0 h, 24 h, 48 h, and 72 h. Magnification: ×200, scale bar = 100 μm. (**D**) Relative expression analysis of E-cadherin and Vimentin in yak RTECs. (**E**,**F**) Protein expression levels of α-SMA and Collagen I in cattle RTECs after 0 h, 24 h, 48 h, and 72 h of hypoxia induction. The original WB images are shown in Figure S2. (**G**,**H**) Protein expression levels of α-SMA and Collagen I in yak RTECs after 0 h, 24 h, 48 h, and 72 h of hypoxia induction. The original WB images are shown in Figure S3. All data are presented as the mean ± SEM, ns = not significant, * *p* < 0.05; ** *p* < 0.01; *** *p* < 0.001.

To determine whether hypoxic responses exhibit species specificity, we subjected RTECs from both cattle and yaks to hypoxic conditions and assessed the expression of epithelial–mesenchymal transition (EMT) markers associated with renal fibrosis. The results indicated that under hypoxia, E-cadherin expression significantly decreased while Vimentin expression significantly increased in bovine RTECs (Figure 2A,B). In contrast, E-cadherin and Vimentin expression in yak RTECs remained unchanged under chronic hypoxic conditions (Figure 2C,D). Furthermore, Western blot analysis revealed that hypoxia notably induced the expression levels of α-SMA and Collagen I in cattle RTECs (Figure 2E,F). Interestingly, compared to the normoxia group, yak RTECs showed increased expression of α-SMA and Collagen I after 24 h of hypoxia; however, with prolonged hypoxia exposure up to 48 and 72 h, their expression returned to normal levels (Figure 2G,H). This suggests that the initial response of yak RTECs to hypoxia may activate certain fibrotic signaling pathways, but subsequently, their hypoxia adaptation mechanisms effectively restore cellular homeostasis, thereby inhibiting further activation of fibrotic pathways. These findings indicate a clear species specificity in hypoxia-induced renal fibrosis, with yaks exhibiting better adaptation to hypoxic environments.

### 3.3. Hypoxia Activates the Mitophagy Pathway in Yak RTECs

**Figure 3:**
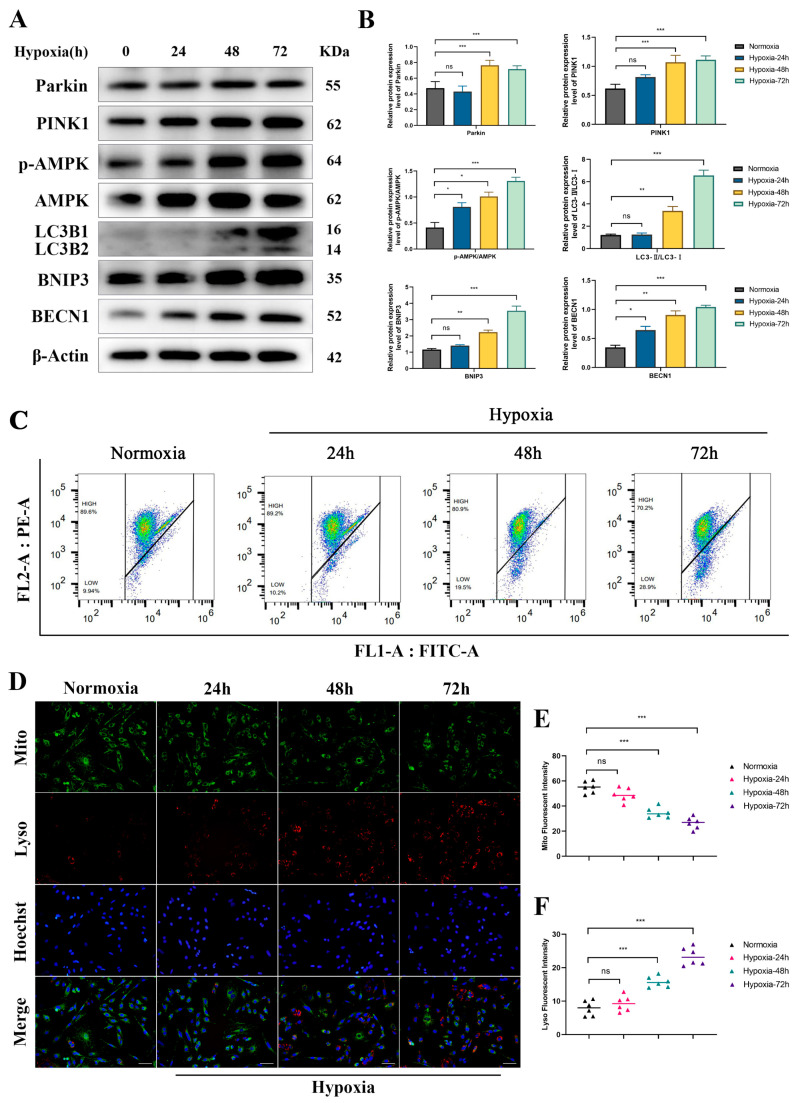
Regulation of Mitophagy under Hypoxia. Cells were exposed to normoxia (21% O_2_) or hypoxia (5% O_2_) for 24 h, 48 h, and 72 h. (**A**) Western blot analysis of Parkin, PINK1, P-AMPK/AMPK, LC3Ⅱ/Ⅰ, and BECN1 expression in RTECs subjected to hypoxia. The original WB images are shown in Figure S4. (**B**) Quantification of the relative expression of Parkin, PINK1, P-AMPK/AMPK, LC3Ⅱ/Ⅰ, BNIP3, and BECN1 in RTECs under hypoxic conditions. (**C**) Changes in mitochondrial membrane potential at different hypoxic time points. (**D**) The effect of hypoxia on the number of mitochondria and lysosomes, with Hoechst staining for nuclei. Magnification: ×200, scale bar = 100 μm. (**E**,**F**) Statistical analysis of mitochondrial and lysosomal counts at different hypoxic time points. All data are presented as the mean ± SEM, ns = not significant, * *p* < 0.05; ** *p* < 0.01; *** *p* < 0.001.

To further investigate the regulatory effect of hypoxia on mitophagy in yak kidneys, we cultured yak RTECs under 5% O_2_ conditions and analyzed the expression levels of mitophagy-related proteins. The results demonstrated that, with prolonged exposure to hypoxia, the expression levels of Parkin, PINK1, P-AMPK/AMPK, LC3Ⅱ/Ⅰ, BNIP3, and BECN1 significantly increased (Figure 3A,B). Mitochondrial membrane potential analysis revealed that hypoxia induced depolarization of the mitochondrial membrane (Figure 3C). Moreover, compared to the normoxic group, the hypoxia-induced group exhibited reduced number of mitochondria, while the number of lysosomes increased (Figure 3D–F). These findings suggest that chronic hypoxia activates the mitophagy pathway in yak RTECs.

### 3.4. Mitophagy Inhibits EMT in Yak RTECs Under Chronic Hypoxia

**Figure 4:**
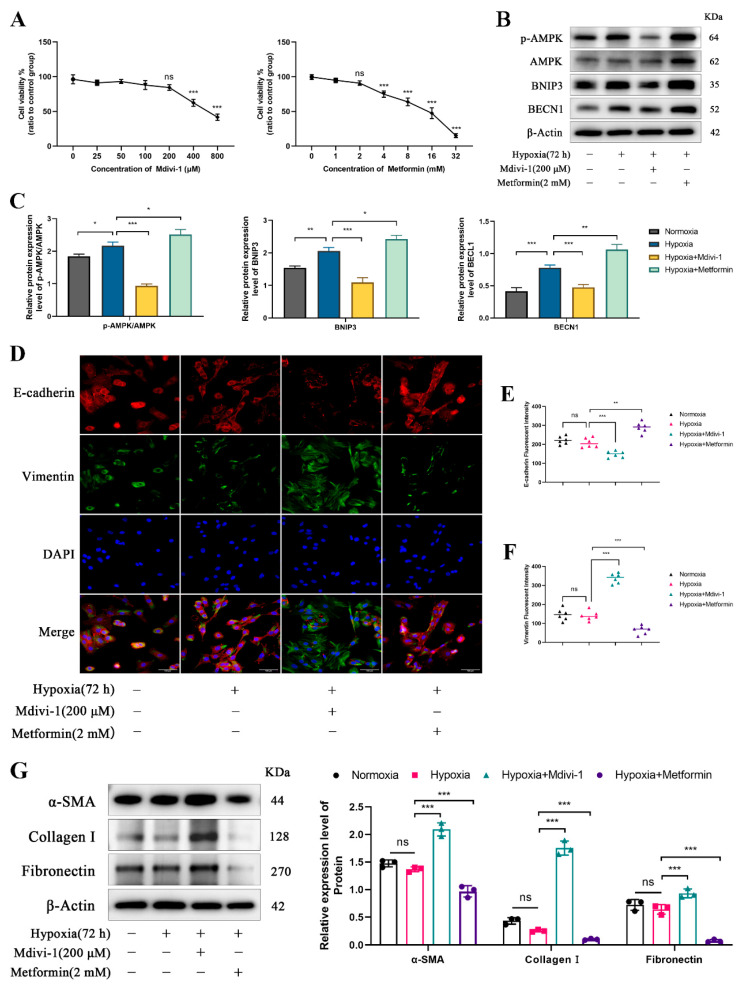
Regulation of renal fibrosis by mitophagy in yaks. (**A**) The optimal concentrations of the mitophagy inhibitor Mdivi-1 and the mitophagy activator Metformin were determined by CCK8 assays for treating yak RTECs. (**B**) Western blot bands for P-AMPK/AMPK, BNIP3, and BECN1 expression in RTECs under different treatments. The original WB images are shown in Figure S5. (**C**) The relative expression levels of P-AMPK/AMPK, BNIP3, and BECN1 proteins. (**D**) Immunofluorescence staining to detect the expression of E-cadherin and Vimentin, with DAPI staining for nuclei. Magnification ×200, scale bar = 100 μm. (**E**,**F**) Analysis of the fluorescence intensity of E-cadherin and Vimentin. (**G**) The relative expression of α-SMA, Collagen I, and Fibronectin proteins in different treatment groups. The original WB images are shown in Figure S6. All data are presented as the mean ± SEM, ns = not significant, * *p* < 0.05; ** *p* < 0.01; *** *p* < 0.001.

EMT of RTECs is a hallmark feature of renal fibrosis. To evaluate the regulatory role of mitophagy in yak renal fibrosis, RTECs were exposed to hypoxia for 72 h, followed by treatment with the mitophagy inhibitor Mdivi-1 or the activator Metformin. Optimal concentrations of Mdivi-1 and Metformin were determined to be 200 μM and 2 mM, respectively (Figure 4A). Western blot analysis revealed that Mdivi-1 significantly inhibited, whereas Metformin promoted the expression of mitophagy-related proteins, including P-AMPK/AMPK, BNIP3, and BECN1 (Figure 4B,C). Immunofluorescence staining showed no significant differences in the expression of E-cadherin and Vimentin between the normoxia group and the chronic hypoxic group (72 h). However, in the Mdivi-1 treatment group, E-cadherin expression was significantly lower, and Vimentin expression was significantly higher compared to the chronic hypoxia group. Conversely, in the Metformin treatment group, E-cadherin expression was significantly higher, whereas Vimentin expression was significantly lower than in the chronic hypoxia group (Figure 4D–F). Further Western blot analysis revealed no significant differences in the expression levels of fibrosis markers, including α-SMA, Collagen I, and Fibronectin, between the normoxia and chronic hypoxic groups. However, Mdivi-1 and Metformin, respectively, upregulated or downregulated the expression of these fibrosis-related proteins (Figure 4G). Collectively, these findings suggest that chronic hypoxia-induced mitophagy effectively inhibits EMT and renal fibrosis in yak RTECs.

### 3.5. Mitophagy Inhibits Ferroptosis in Yak RTECs

**Figure 5:**
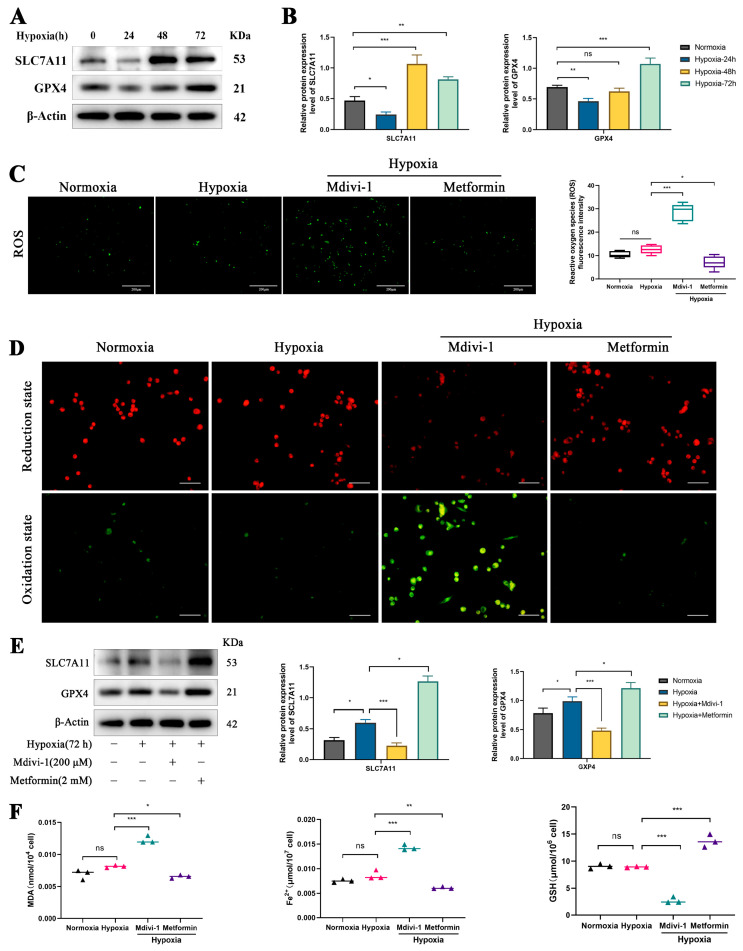
The regulatory role of mitophagy in ferroptosis under chronic hypoxia. (**A**) Western blot bands of SLC7A11 and GPX4 after hypoxia induction for 24 h, 48 h, and 72 h. The original WB images are shown in Figure S7. (**B**) Relative protein expression levels of SLC7A11 and GPX4 under different durations of hypoxia. (**C**) ROS expression levels under chronic hypoxia and after treatment with Mdivi-1 and Metformin. Magnification ×100, scale bar = 200 μm. (**D**) Lipid oxidation was detected using the bodipy assay kit. Red fluorescence indicates the reduction state of lipids, while green fluorescence indicates the oxidation state in RTECs. Magnification ×200, scale bar = 100 μm. (**E**) Relative protein expression levels of SLC7A11 and GPX4 under chronic hypoxia and after treatment with Mdivi-1 and Metformin. The original WB images are shown in Figure S8. (**F**) Measurements of MDA, Fe^2+^, and GSH levels in different treatment groups. All data are presented as the mean ± SEM, ns = not significant, * *p* < 0.05; ** *p* < 0.01; *** *p* < 0.001.

Ferroptosis is one of the major mechanisms for hypoxia-induced renal fibrosis. Therefore, we hypothesize that mitophagy activation in yaks under chronic hypoxia protects against hypoxia-induced renal fibrosis by inhibiting ferroptosis. To test this hypothesis, we analyzed the expression levels of key ferroptosis-related proteins under different hypoxia durations. The results showed that at 24 h of hypoxia, the expression of SLC7A11 and GPX4 was downregulated, but as the hypoxia duration increased, the expression levels of SLC7A11 and GPX4 significantly increased, indicating that ferroptosis was inhibited under chronic hypoxia (Figure 5A,B). Using the mitophagy inhibitors (Mdivi-1) and activators (Metformin) to treat yak RTECs under chronic hypoxia, we found that Mdivi-1 promoted ROS production and lipid oxidation, whereas Metformin inhibited these processes (Figure 5C,D). Additionally, in the Mdivi-1 group, the expression levels of SLC7A11 and GPX4 and the GSH content decreased, while MDA and Fe^2+^ levels increased (Figure 5E,F). In contrast, in the Metformin group, the expression levels of SLC7A11 and GPX4 and the GSH content increased, while MDA and Fe^2+^ levels decreased (Figure 5E,F). These findings indicate that the activation of mitophagy under chronic hypoxia inhibits ferroptosis in yak RTECs.

### 3.6. Regulatory Role of Ferroptosis in Yak Renal Fibrosis

**Figure 6:**
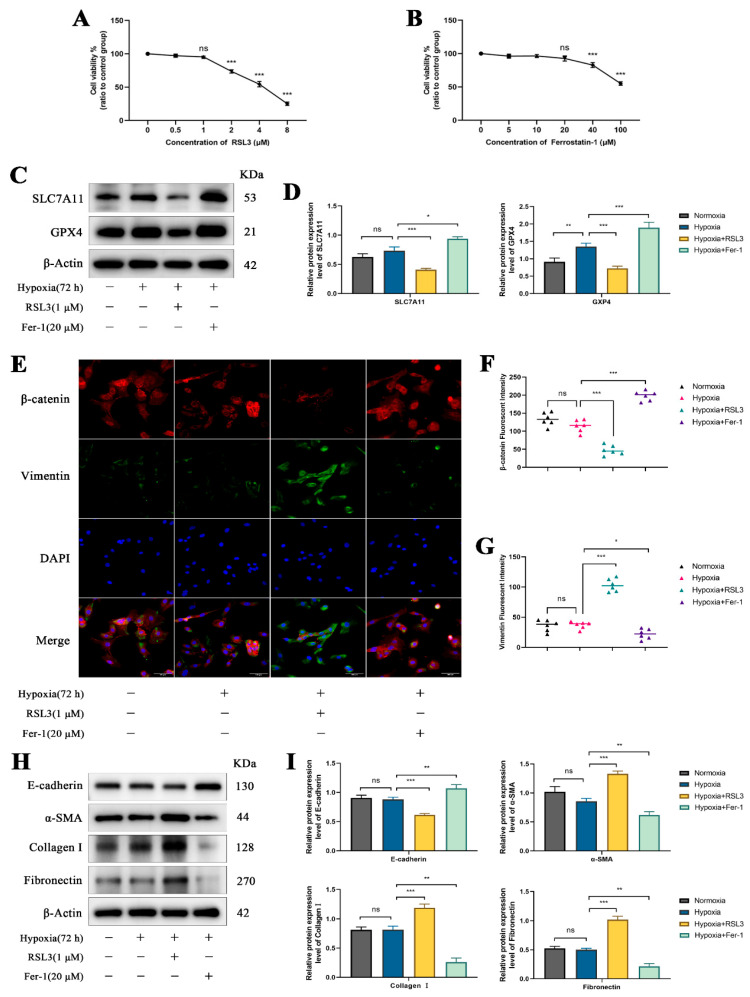
Regulation of ferroptosis in yak renal fibrosis under chronic hypoxia. (**A**,**B**) The optimal drug concentrations of RSL3 and Ferrostatin-1 were determined using CCK8 assays. (**C**,**D**) Relative protein expression levels of SLC7A11 and GPX4 under chronic hypoxia and after treatment with RSL3 and Ferrostatin-1. The original WB images are shown in Figure S9. (**E**) Immunofluorescence staining for the epithelial marker β-catenin and the fibroblast marker Vimentin, with DAPI staining for nuclei. Magnification ×200, scale bar = 100 μm. (**F**,**G**) Fluorescence intensity analysis of β-catenin and Vimentin under chronic hypoxia and after treatment with RSL3 and Ferrostatin-1. (**H**) Western blot bands for E-cadherin, α-SMA, Collagen I, and Fibronectin. The original WB images are shown in Figure S10. (**I**) Relative protein expression levels of E-cadherin, α-SMA, Collagen I, and Fibronectin in the different treatment groups. All data are presented as the mean ± SEM, ns = not significant, * *p* < 0.05; ** *p* < 0.01; *** *p* < 0.001.

To elucidate the regulatory role of ferroptosis in yak renal fibrosis, ferroptosis activator RSL3 and inhibitor Ferrostatin-1 were used to treat RTECs after chronic hypoxia (72 h). Drug concentration assays revealed that the optimal concentrations for RSL3 and Ferrostatin-1 were 1 μM and 20 μM, respectively (Figure 6A,B). Western blot analysis showed that SLC7A11 and GPX4 expression levels decreased in the RSL3 group, while Ferrostatin-1 treatment increased their expression, indicating that RSL3 and Ferrostatin-1 effectively regulate ferroptosis (Figure 6C,D). Immunofluorescence analysis revealed that RSL3 inhibited the expression of the epithelial marker β-catenin and promoted the expression of the fibroblast marker Vimentin. Conversely, Ferrostatin-1 promoted β-catenin expression and inhibited Vimentin expression (Figure 6E–G). Additionally, RSL3 inhibited E-cadherin expression and promoted the expression of fibrotic proteins α-SMA, Collagen I, and Fibronectin. In contrast, Ferrostatin-1 upregulated E-cadherin expression while downregulating α-SMA, Collagen I, and Fibronectin (Figure 6H,I). These findings suggest that ferroptosis activation exacerbates fibrosis progression, whereas its inhibition mitigates fibrosis.

### 3.7. Mitophagy Alleviates Renal Fibrosis by Inhibiting Ferroptosis

**Figure 7:**
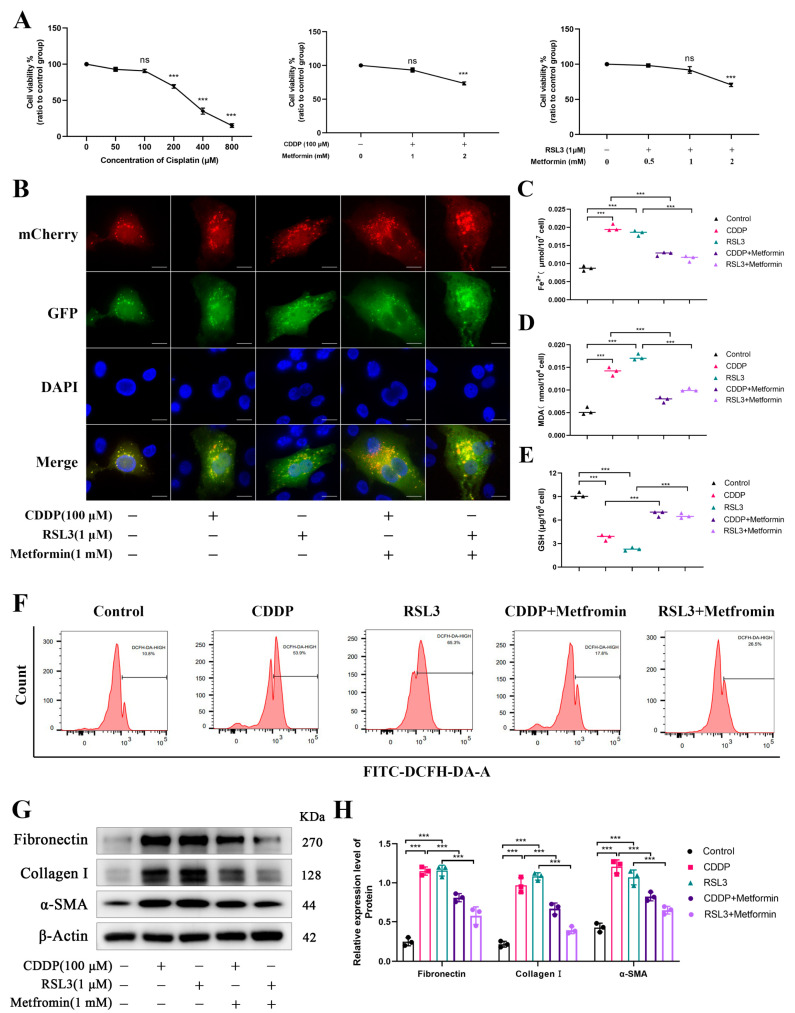
Mitophagy alleviates renal fibrosis by inhibiting ferroptosis. (**A**) CCK8 assay used to determine the optimal concentration of CDDP for renal fibrosis induction, as well as the optimal concentrations of CDDP/RSL3 in combination with Metformin. (**B**) Expression of autophagosome under CDDP, RSL3, Metformin, and CDDP/RSL3 treatment, with DAPI staining for nuclei. Magnification ×600, scale bar = 25 μm. (**C**–**E**) Expression levels of Fe^2^⁺, MDA, and GSH in RTECs treated with CDDP, RSL3, Metformin, and CDDP/RSL3. (**F**) Flow cytometry analysis of ROS levels in different treatment groups. (**G**) Western blot bands of Fibronectin, Collagen I, and α-SMA. The original WB images are shown in Figure S11. (**H**) Relative protein expression of Fibronectin, Collagen I, and α-SMA in response to CDDP, RSL3, Metformin and CDDP/RSL3 treatment. All data are presented as the mean ± SEM, ns = not significant, *** *p* < 0.001.

Based on the above findings, we further explored the roles of mitophagy and ferroptosis in renal fibrosis. We induced yak RTECs with either cisplatin (CDDP, a renal fibrosis inducer) or RSL3, followed by treatment with Metformin to evaluate its therapeutic effects on fibrosis. Drug concentration assays identified optimal working concentrations of 100 μM for CDDP, and a combined treatment concentration of 1 mM for metformin when used with either CDDP or RSL3 (Figure 7A). Ad-mCherry-GFP-LC3B analysis showed that both CDDP and RSL3 significantly promoted autophagosome formation (Figure 7B). Concurrently, levels of Fe^2^⁺, MDA, and ROS increased, while GSH expression decreased, indicating the activation of both mitophagy and ferroptosis in the renal fibrosis model (Figure 7C–F). Metformin treatment in CDDP or RSL3-induced fibrosis reduced α-SMA, Collagen I, and Fibronectin expression levels, suggesting that activating mitophagy can alleviate renal fibrosis (Figure 7G,H). Notably, metformin decreased Fe^2^⁺, MDA, and ROS levels, while enhancing GSH expression in the CDDP/RSL3-treated groups, further supporting the notion that activation of mitophagy inhibits ferroptosis in renal fibrosis (Figure 7C–F). These findings suggest that mitophagy may alleviate renal fibrosis by suppressing ferroptosis pathways.

## 4. Discussion

Renal fibrosis is a critical pathological feature in the progression of CKD, regulated by various factors, including hypoxia, inflammation, oxidative stress, and epithelial cell injury [2]. Among these, hypoxia plays a crucial role in driving renal fibrosis. In a hypoxic environment, insufficient oxygen supply to renal tissues activates hypoxia signaling pathways, which subsequently upregulate the expression of multiple pro-fibrotic factors [20,21]. These factors not only promote fibroblast activation but also induce aberrant accumulation of ECM, thereby exacerbating fibrotic progression [22]. However, the impact of hypoxia on the kidneys may differ depending on species-specific adaptive mechanisms. Yaks, which have long inhabited high-altitude hypoxic environments, yet no studies have shown that hypoxia induces kidney disease in yaks. In this study, by comparing the renal tissue structures of cattle and yaks, we observed that yaks have a significantly higher number of renal tubules than cattle. Renal tubules play a crucial role in water reabsorption and in regulating fluid and acid-base balance. High-altitude environments are often accompanied by low temperatures and dryness, which can lead to water loss. Therefore, we speculate that an increased number of renal tubules may help yaks more effectively retain body fluids and electrolytes, thereby maintaining fluid balance and normal physiological functions. Additionally, collagen volume analysis indicated no significant difference in volume fraction between the two species. This result suggests that yaks may have developed anti-fibrotic mechanisms during prolonged hypoxic adaptation. However, the molecular mechanisms by which yaks avoid hypoxia-induced renal fibrosis have not been reported.

In our previous research, we identified mitophagy as a pivotal regulatory mechanism for maintaining mitochondrial homeostasis in yaks [15]. Notably, mitochondrial dysfunction can lead to RTEC injury and accelerate the progression of renal fibrosis [23,24]. Therefore, we hypothesized that yaks might activate the mitophagy pathway in high-altitude hypoxic environments to protect their kidneys from fibrotic damage. To validate this hypothesis, we compared the expression levels of mitophagy-related proteins in the kidneys of cattle and yaks. The results demonstrated a significant upregulation of P-AMPK/AMPK, Parkin, PINK1, LC3 Ⅱ/Ⅰ, and BECN1, along with a downregulation of P-mTOR/mTOR in yak kidneys under hypoxia. BNIP3 has been widely recognized as a key regulator of mitophagy [25], primarily by disrupting the interaction between Bcl-2 and BECN1, thereby releasing BECN1 to promote autophagosome formation [26]. Meanwhile, hypoxia inhibits oxidative phosphorylation, reducing ATP levels and activating AMPK. Activated AMPK suppresses the mTOR pathway, relieving its inhibitory effect on mitophagy [27,28]. Collectively, these findings indicate that hypoxia activates the mitophagy pathway in the yak kidney, potentially playing a protective role in the adaptation of yak kidneys to hypoxic environments and inhibiting fibrosis progression.

EMT is a critical pathological process in renal fibrosis. Under hypoxic conditions, EMT causes RTECs to gradually lose their epithelial phenotype and acquire migratory mesenchymal characteristics [29]. This process is characterized by a decrease in E-cadherin expression and an increase in Vimentin, α-SMA, and FN1 expression. Our study found that under chronic hypoxia, mitophagy activation effectively inhibited the expression of Vimentin, α-SMA, Collagen Ⅰ, and FN1, suggesting that mitophagy may play a protective role in EMT progression and renal fibrosis. This result is consistent with previous studies. Multiple studies have confirmed that mitophagy not only alleviates hypoxia-induced renal fibrosis but also has therapeutic effects on other kidney diseases. For instance, Mdivi-1, a mitochondrial inhibitor, can reduce the expression levels of PINK1, PARK2, and LC3II, activate the TGF-β1 signaling pathway in HK-2 cells under hypoxic conditions, thereby exacerbating renal fibrosis [30]. Additionally, mitophagy can inhibit the expression of fibrosis-related proteins through the AMPK/mTOR signaling pathway, ultimately preventing the progression of renal fibrosis [31]. In studies on ischemia/reperfusion (I/R)-induced acute kidney injury (AKI), it was found that HIF-1α-BNIP3 can induce mitophagy in HK-2 cells, inhibit apoptosis and ROS production in AKI, thereby exerting a protective effect on the kidney [25]. Another study also showed that mitophagy inhibits the occurrence of AKI by reducing the activation of the NOD-like receptor protein 3 (NLRP3) inflammasome, which plays a crucial role in the pathogenesis of AKI [32].

Ferroptosis is an iron-dependent form of cell death, characterized by elevated intracellular ROS levels, lipid peroxidation accumulation, and disruption of the GSH/GPX4 antioxidant system [33]. Under hypoxic conditions, HIF-1α inhibits the expression of SLC7A11, reducing cellular cysteine uptake, which subsequently lowers GSH levels and inhibits GPX4 activity [34]. This change prevents the effective clearance of lipid peroxides, ultimately inducing ferroptosis. Concurrently, oxidative stress triggered by ferroptosis can activate the NF-κB signaling pathway, leading to the release of pro-inflammatory factors such as interleukin-6 (IL-6) and tumor necrosis factor-alpha (TNF-α). These inflammatory factors further stimulate the TGF-β1 pathway, inducing EMT and thereby accelerating fibrosis progression. However, our study revealed that yak RTECs did not exhibit an enhanced trend of ferroptosis under chronic hypoxia and maintained normal expression of SLC7A11 and GPX4. Notably, when ferroptosis was modulated in yak RTECs using RSL3 (ferroptosis inducer) or Ferrostatin-1 (ferroptosis inhibitor), EMT and fibrosis were, respectively, promoted or inhibited. These findings suggest that the hypoxic adaptation mechanisms in yaks effectively suppress hypoxia-induced ferroptosis, thereby mitigating renal fibrosis progression.

In the recent years, increasing evidence has highlighted a close relationship between ferroptosis and mitophagy in the regulation of renal fibrosis. Studies have demonstrated that during ferroptosis, mitochondria undergo morphological changes, including shrinkage, increased membrane density, cristae disappearance, and outer membrane rupture [35]. These alterations lead to mitochondrial dysfunction and excessive ROS accumulation, further exacerbating ferroptosis. However, mitophagy can clear damaged mitochondria, reduce ROS production, and prevent GPX4 inactivation and lipid peroxidation accumulation, thereby effectively inhibiting ferroptosis [17]. Our study similarly revealed that under chronic hypoxia, mitophagy activation significantly reduced ROS and lipid peroxides while upregulating SLC7A11 and GPX4 expression. However, recent research has proposed different perspectives, suggesting that mTOR inhibition can enhance mitophagy-mediated GPX4 degradation, thereby promoting ferroptosis in bladder cancer cells [36]. Additionally, the AMPK pathway can phosphorylate BECN1 at Ser90/93/96 sites, facilitating the formation of the BECN1-SLC7A11 complex, which induces lipid peroxidation and ferroptosis [37]. These studies underscore the complex role of mitophagy in ferroptosis. Nevertheless, our study supports the view that mitophagy inhibits ferroptosis. In our study, we found that in a cisplatin-induced renal fibrosis model, both mitophagy and ferroptosis pathways were activated. However, either activating mitophagy or inhibiting ferroptosis effectively suppressed fibrosis-related protein expression induced by cisplatin. Furthermore, mitophagy activation significantly reduced ROS and MDA levels while promoting GSH expression in the fibrosis model. Therefore, our study suggests that under chronic hypoxia, mitophagy serves as an adaptive protective mechanism that protects yak kidneys from hypoxia-induced fibrosis by inhibiting ferroptosis.

In addition to mitophagy activation, other antifibrotic mechanisms may play significant roles in mitigating hypoxia-induced renal fibrosis. Studies have shown that HIF-2α activated under hypoxic conditions can improve tubulointerstitial oxygenation, promote angiogenesis, and inhibit the expression of fibrosis-related genes, thereby reducing tubulointerstitial injury and renal fibrosis [38,39]. Moreover, in a UUO of renal fibrosis in mice, erythropoietin (EPO) treatment was found to suppress the accumulation of myofibroblasts and the expression of α1(I) collagen mRNA, ultimately alleviating the progression of renal fibrosis [40]. Interestingly, our previous work demonstrated that hypoxia significantly induces EPO expression in yak kidneys [4]. Furthermore, enhanced glycolysis under hypoxia represents another important mechanism for reducing fibrosis. Research indicates that increased glycolysis leads to hyperacetylation of mitochondrial proteins, and the deacetylation of the pyruvate dehydrogenase complex (PDC) at lysine 385 can be significantly reversed, thereby improving renal fibrosis [41]. However, whether these mechanisms are involved in protecting yaks from hypoxia-induced renal fibrosis requires further investigation.

## 5. Conclusions

In summary, the high-altitude hypoxic environment does not induce renal fibrosis in yaks. Further research indicates that chronic hypoxia activates the mitophagy pathway. By modulating mitophagy under chronic hypoxia, we found that mitophagy inhibits the processes of renal fibrosis. Additionally, this study demonstrates that ferroptosis is a significant mechanism inducing renal fibrosis, and that mitophagy can protect yaks from hypoxia-induced renal fibrosis by inhibiting ferroptosis.

## Data Availability

The data that support the study findings are available upon request.

## Supplementary Materials

The following supporting information can be downloaded at: https://www.mdpi.com/article/10.3390/biom15040556/s1, File S1: Western blot original images.

## Abbreviations

The following abbreviations are used in this manuscript:

RTECs	Renal Tubular Epithelial Cells
CKD	Chronic kidney disease
HIF-1α	Hypoxia-Inducible Factor 1-alpha
AMPK	AMP-Activated Protein Kinase
EMT	Epithelial–Mesenchymal Transition
ROS	Reactive Oxygen Species
α-SMA	Alpha-Smooth Muscle Actin
MDA	Malondialdehyde
GSH	Glutathione
SCL7A11	Solute Carrier Family 7 Member 11
GPX4	Glutathione Peroxidase 4
