# Supplementary material for: Mechanism of Mitophagy to Protect Yak Kidney from Hypoxia-Induced Fibrosis Damage by Regulating Ferroptosis Pathway
Source: Biomolecules. 2025 Apr 9;15(4):556. doi: 10.3390/biom15040556 (PMC12025222; doi:10.3390/biom15040556)
Supplement: Supplementary file 1 [file biomolecules-15-00556-s001.zip › biomolecules-3504249-supplementary.pdf]

Supplementary Figures

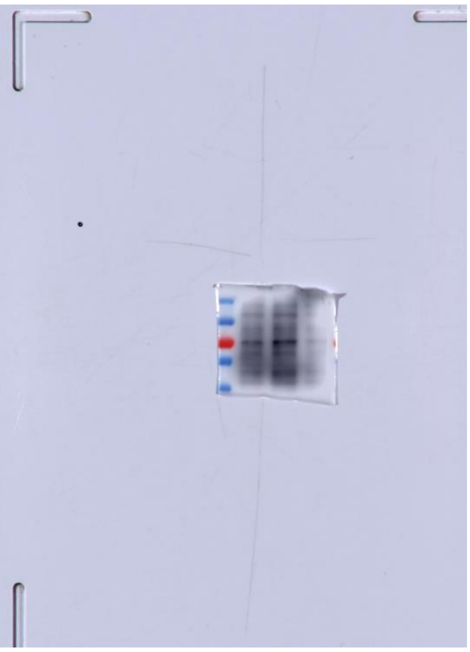

P-AMPK

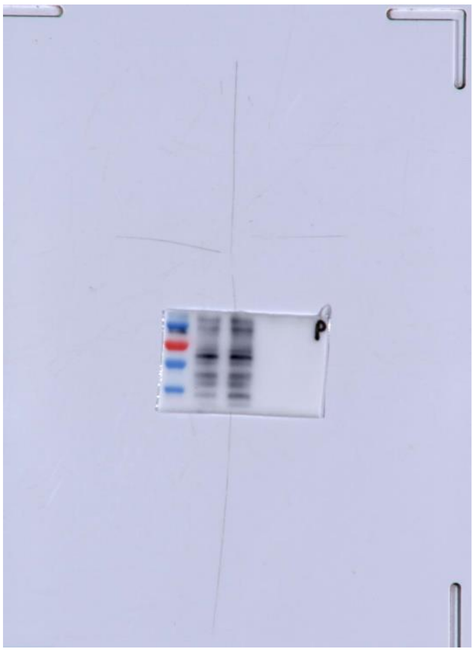

AMPK

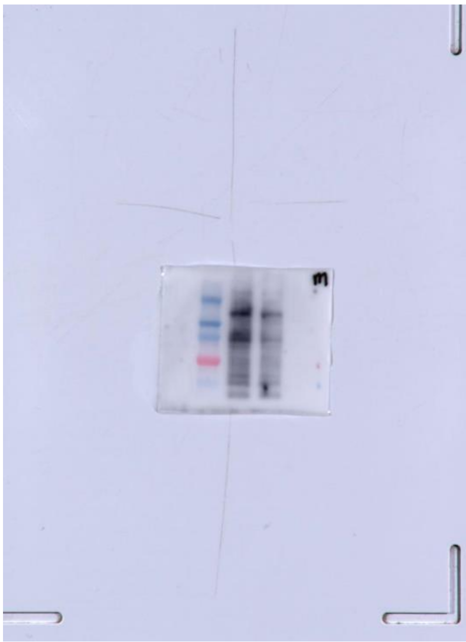

P-mTOR

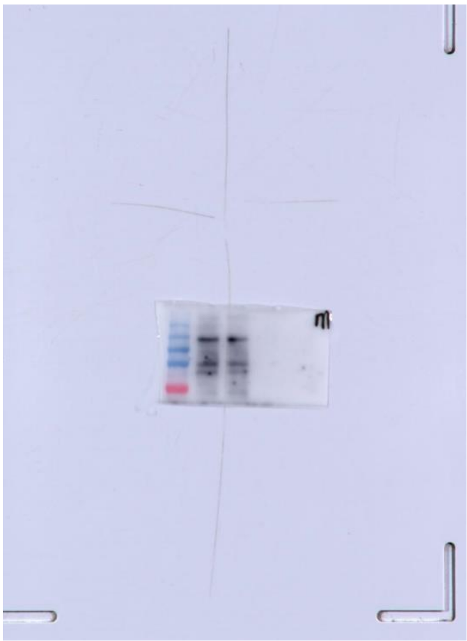

mTOR

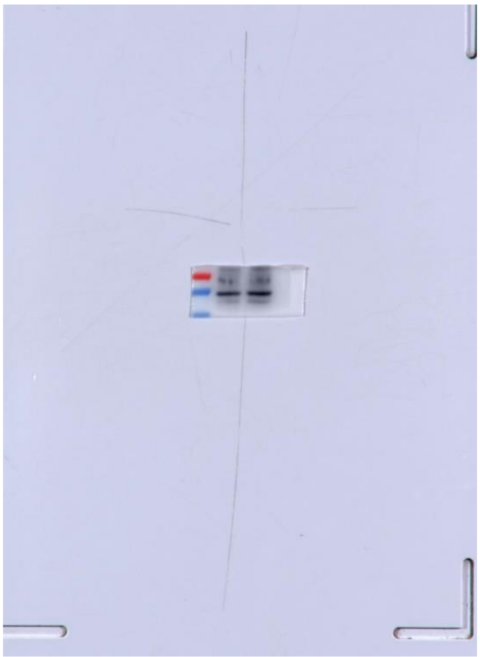

Parkin

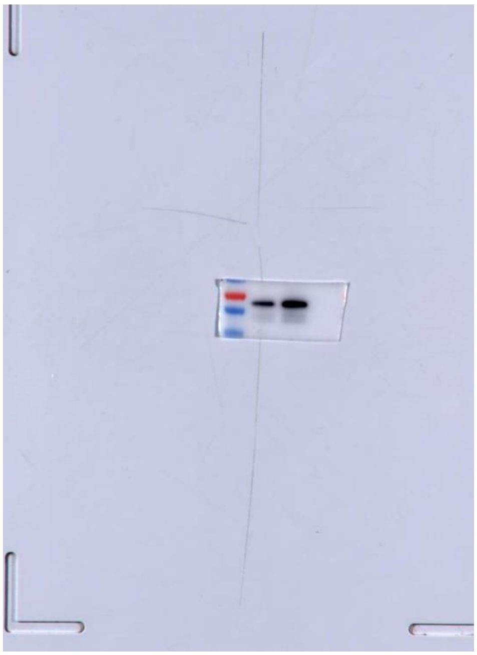

PINK1

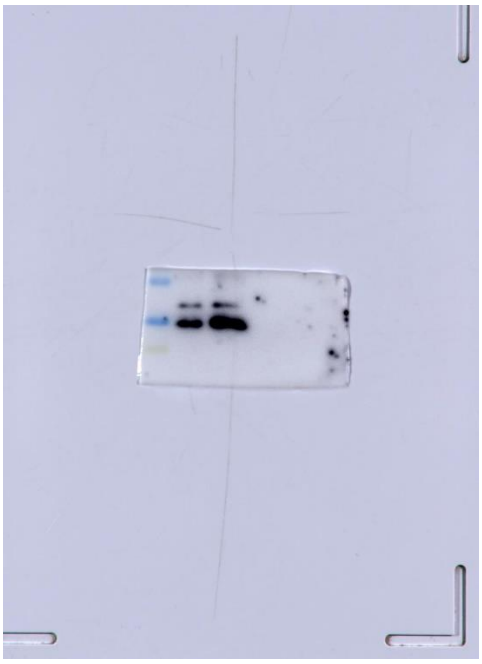

LC3

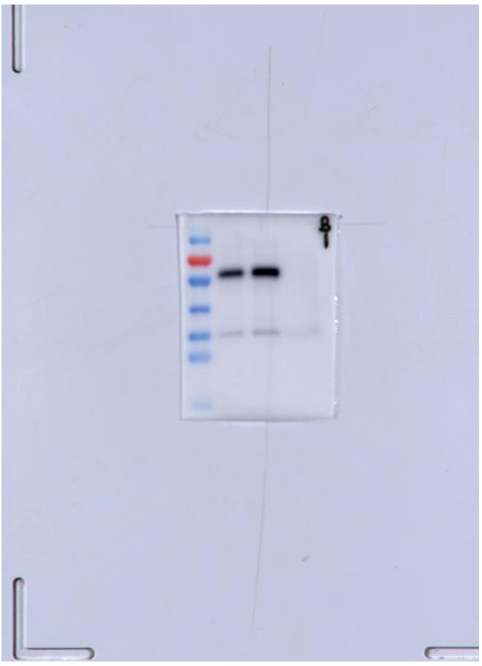

BECN1

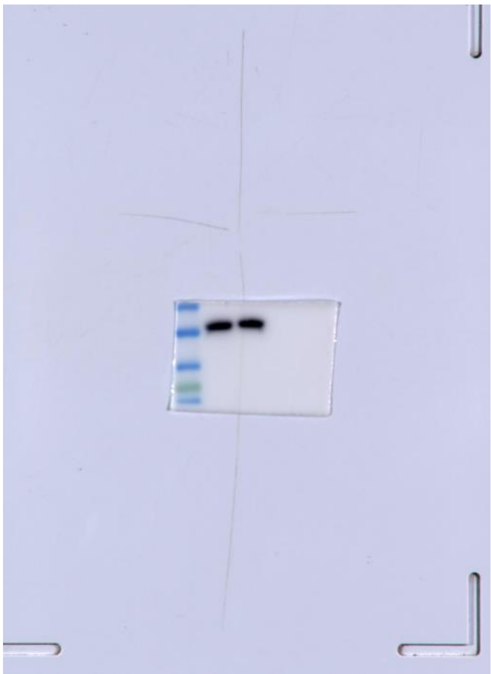

β-Actin

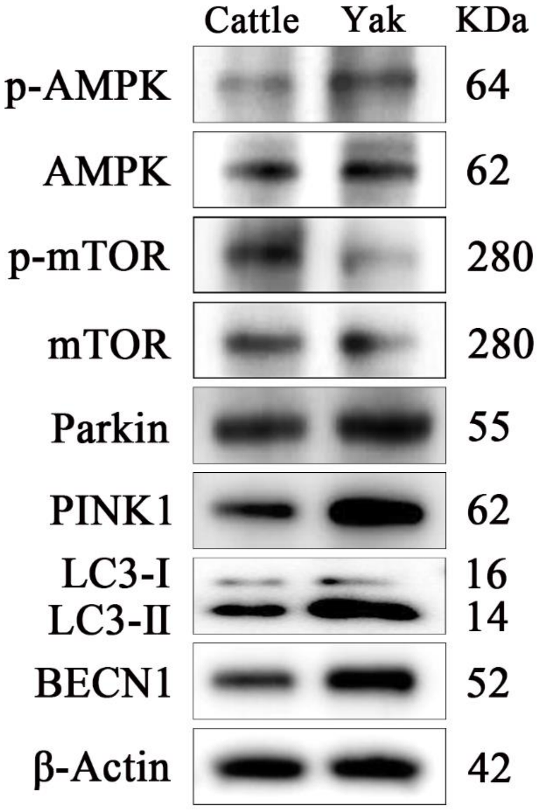

Figure S1. The original WB images of Figure 1F.

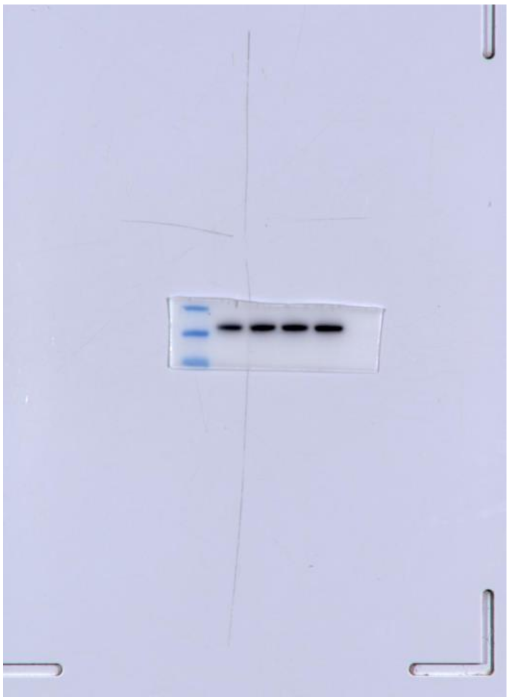
Cattle-α-SMA

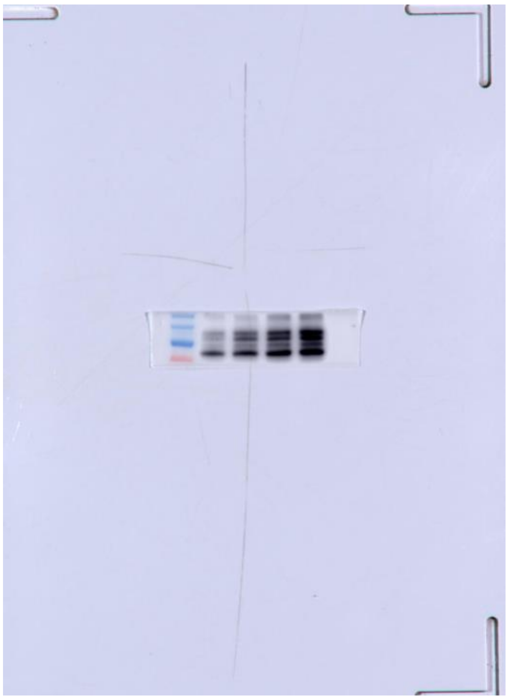
Cattle-Collagen I

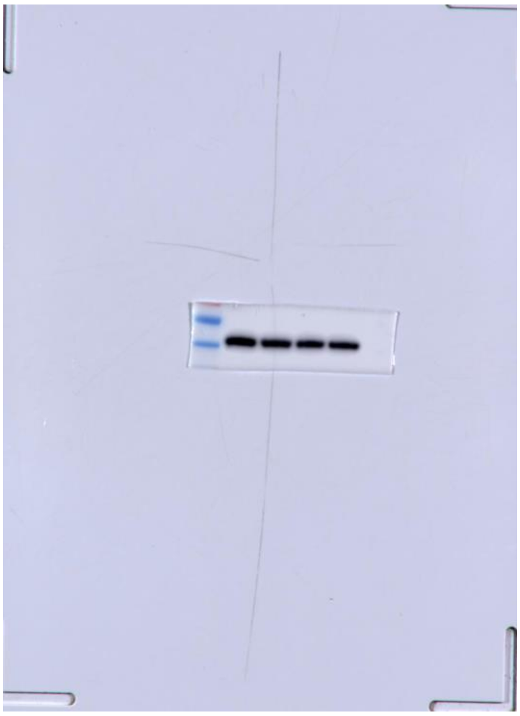
Cattle-β-Actin

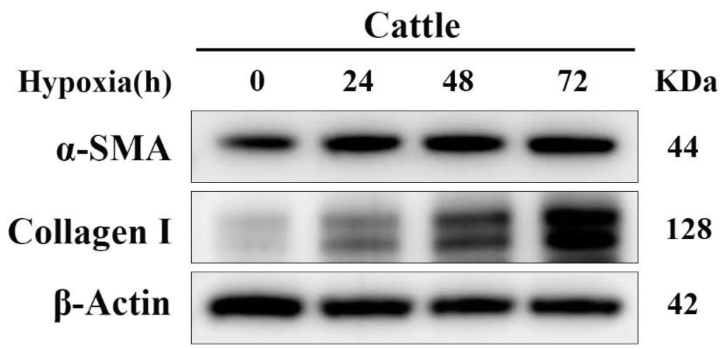

Figure S2. The original WB images of Figure 2E.

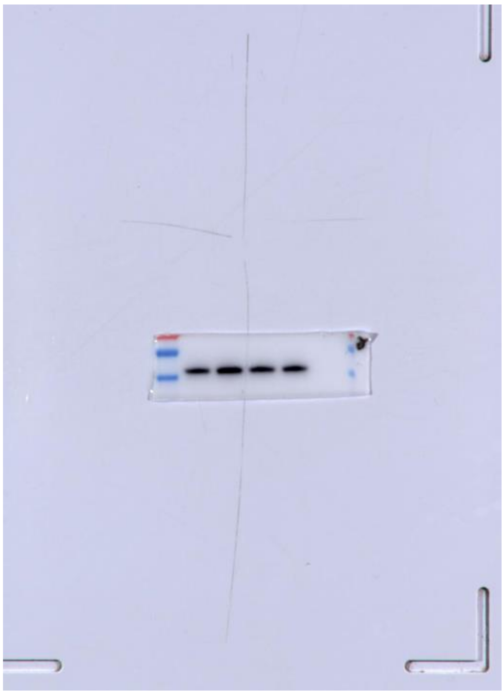

Yak-α-SMA

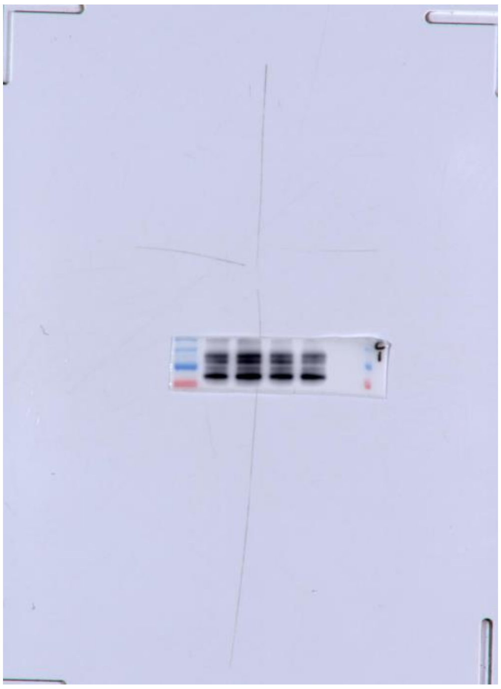

Yak-Collagen I

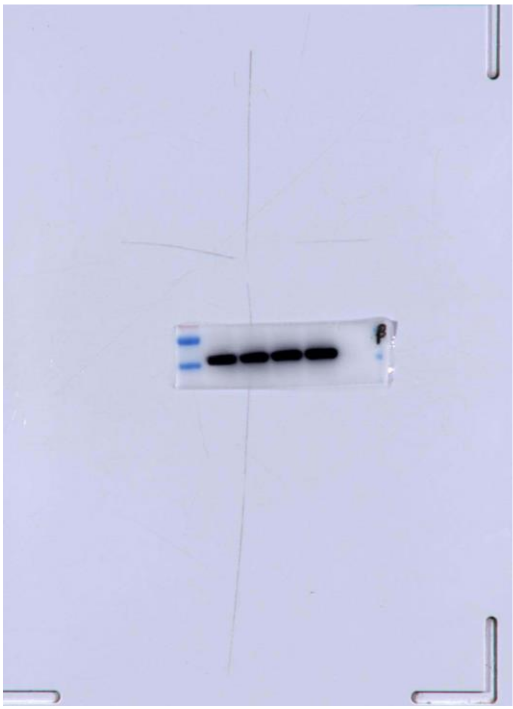

Yak-β-Actin

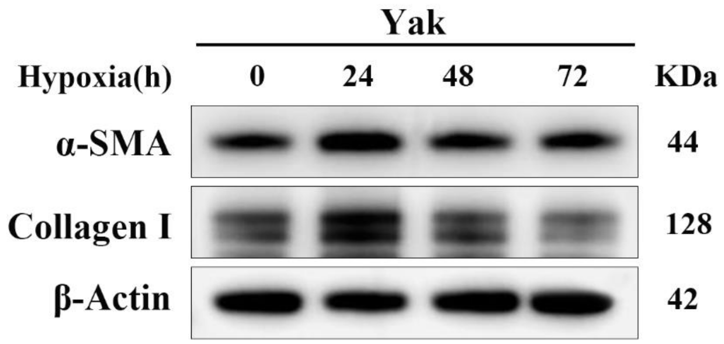

Figure S3. The original WB images of Figure 2G.

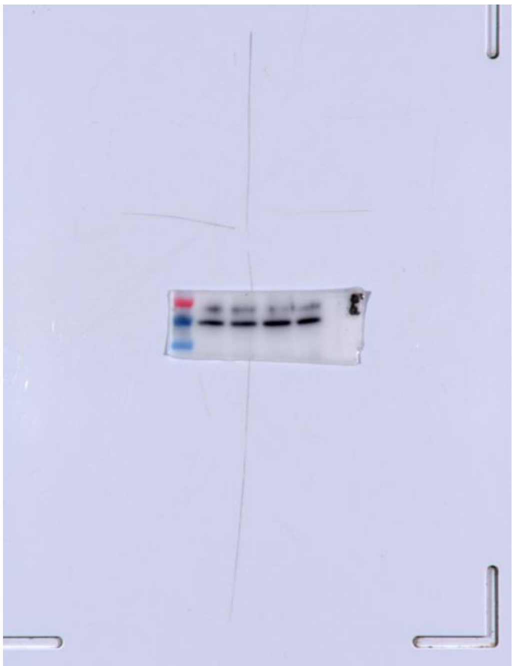

Parkin

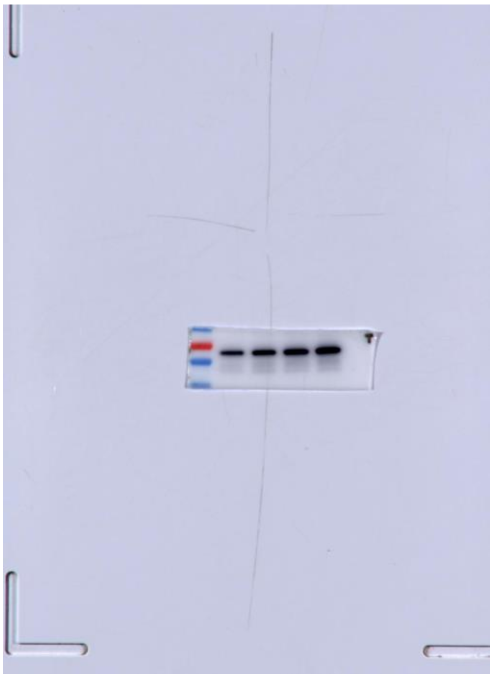

PINK1

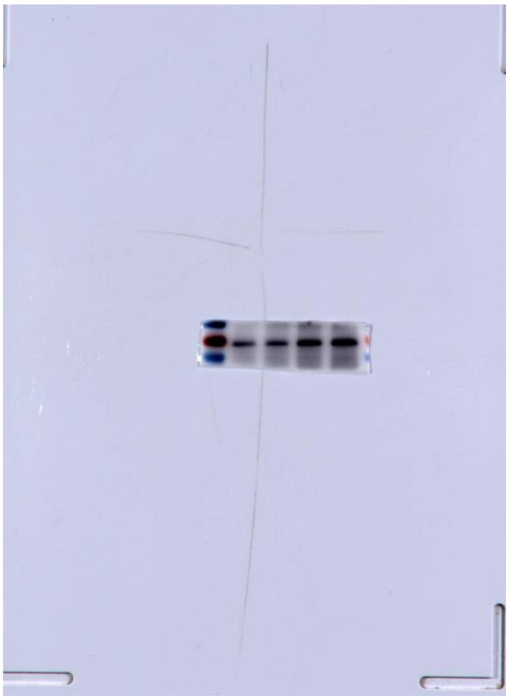

P-AMPK

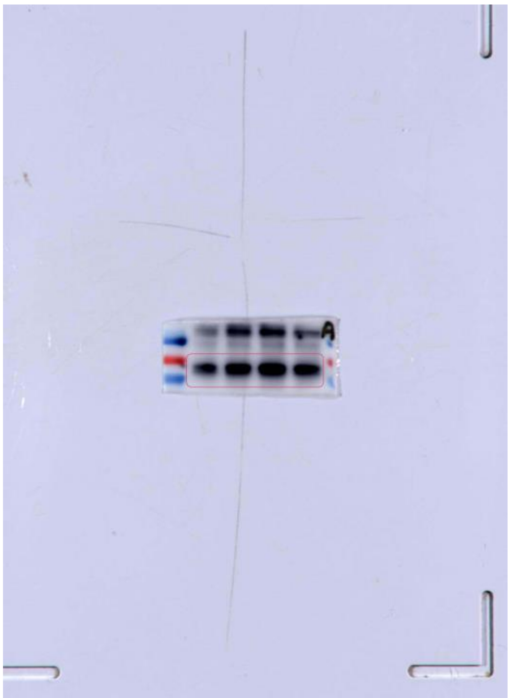

AMPK

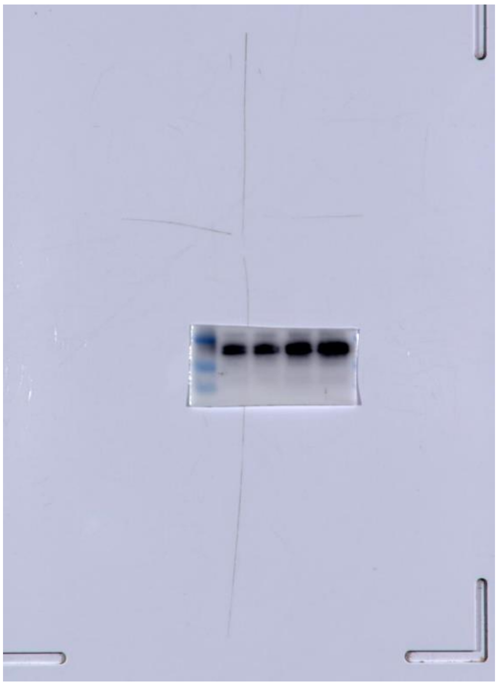

BNIP3

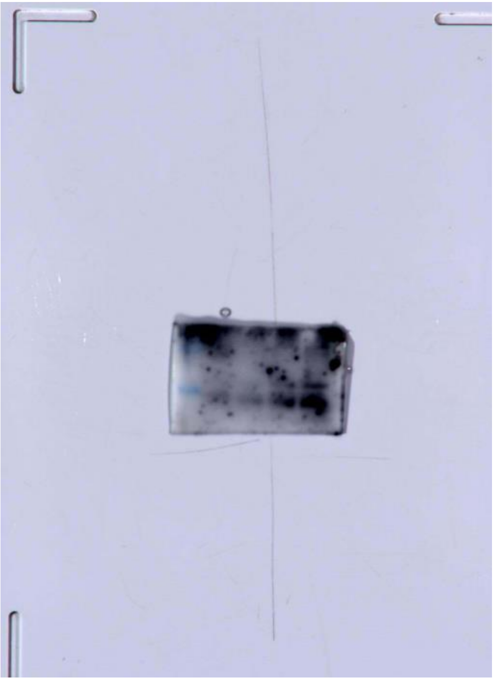

LC3

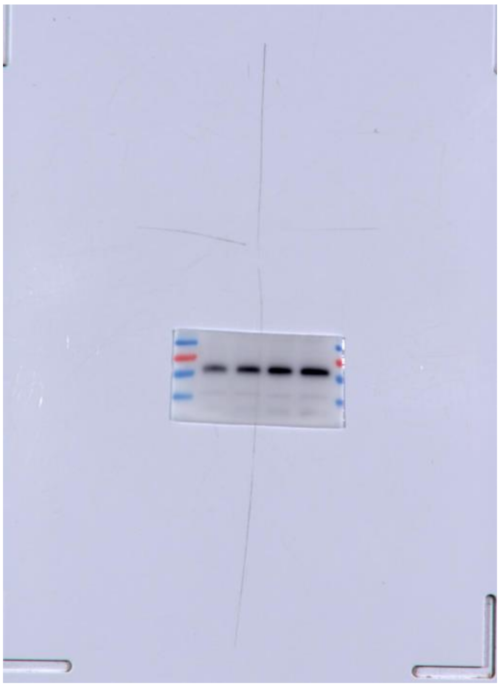

BECN1

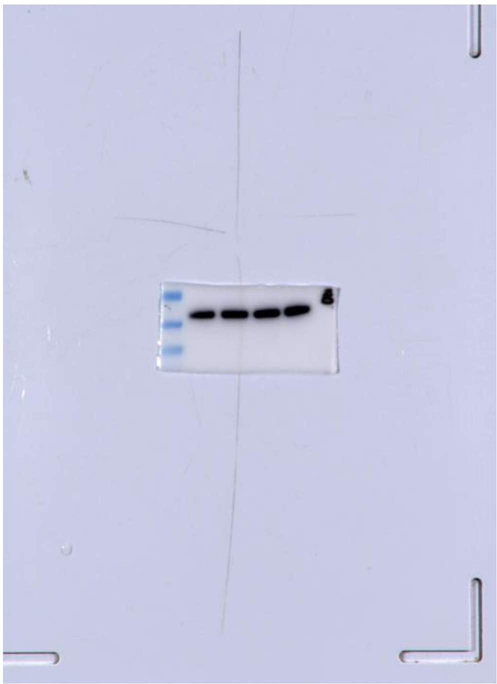

β-Actin

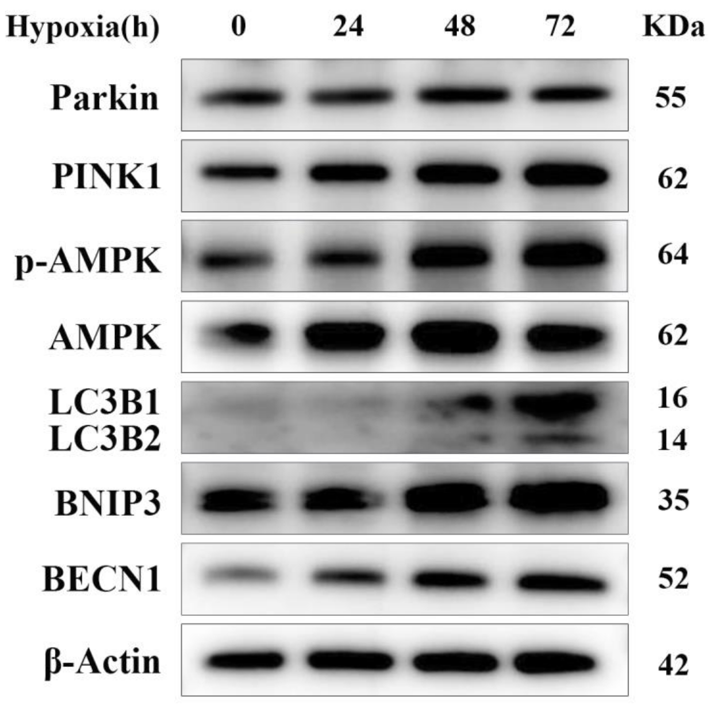

Figure S4. The original WB images of Figure 3A.

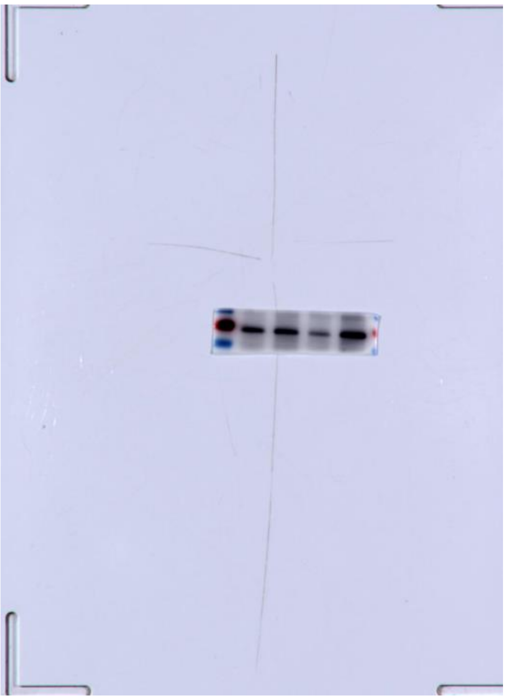

P-AMPK

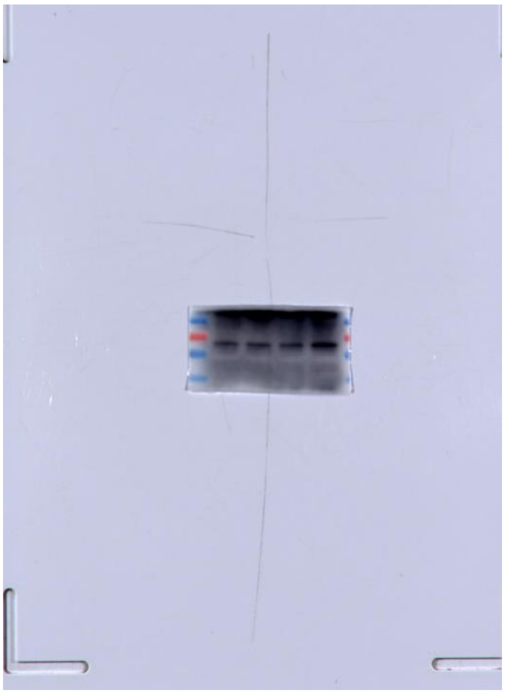

AMPK

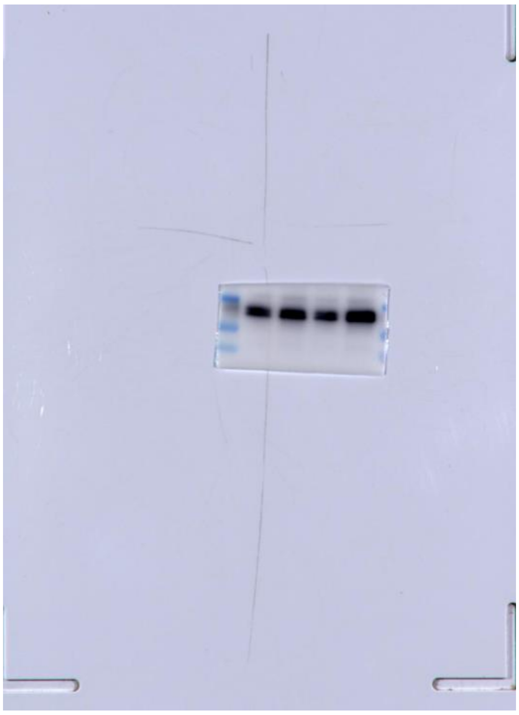

BNIP3

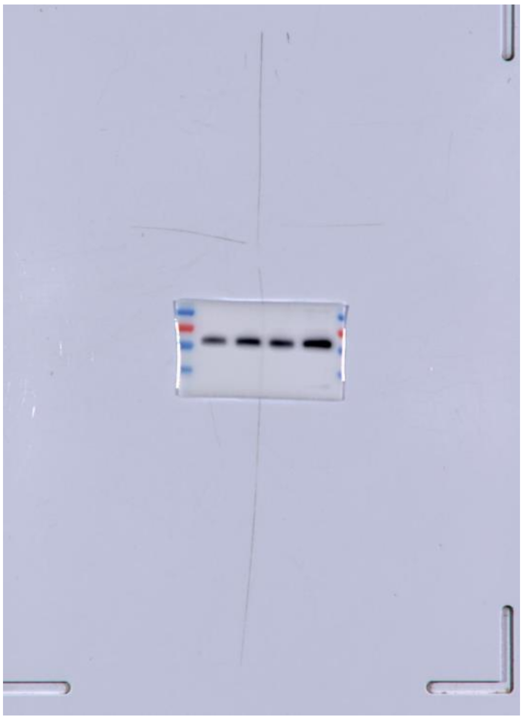

BECN1

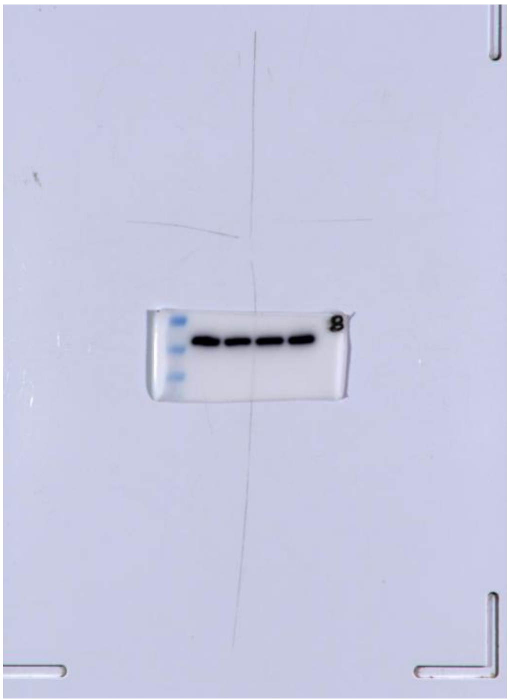

β-Actin

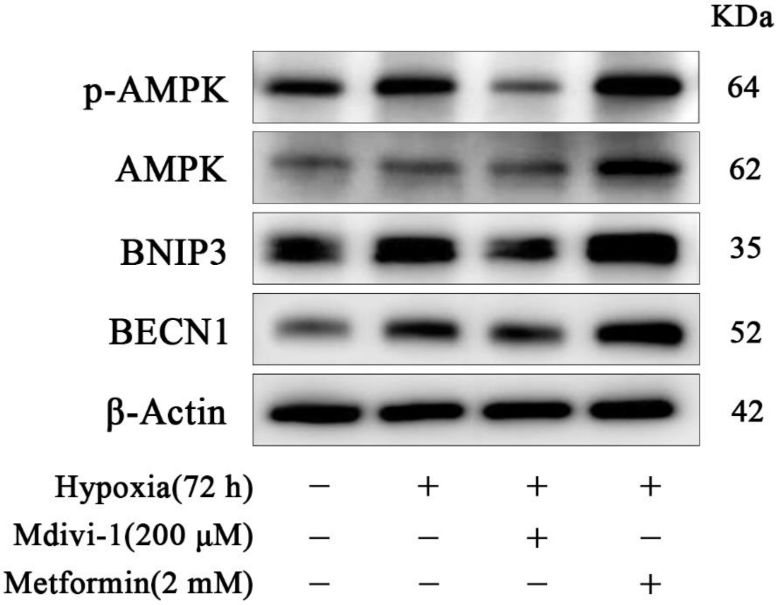

Figure S5. The original WB images of Figure 4B.

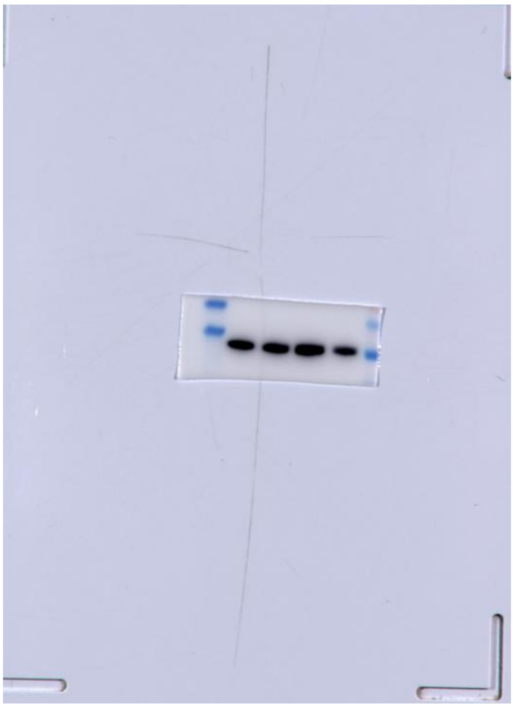

α-SMA

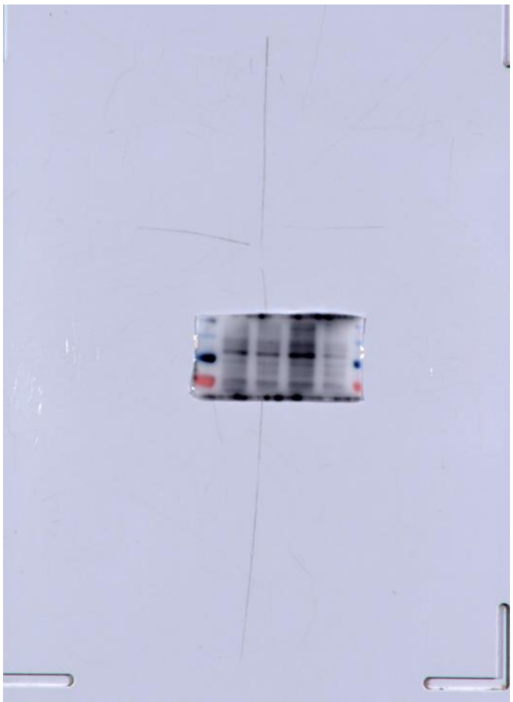

Collagen I

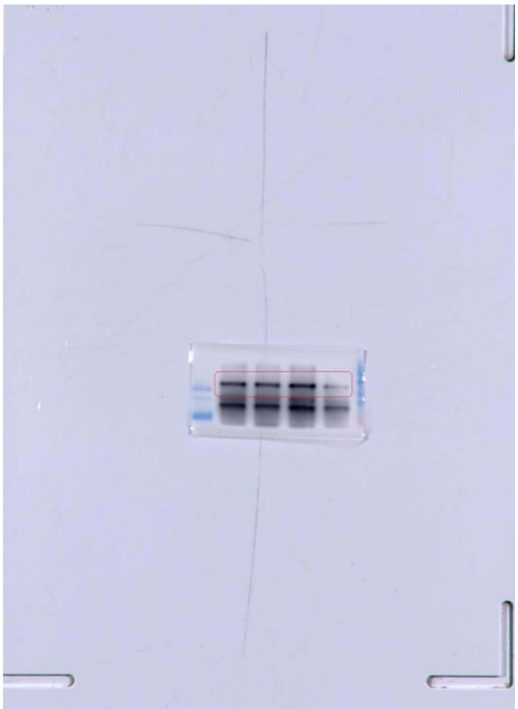

Fibronectin

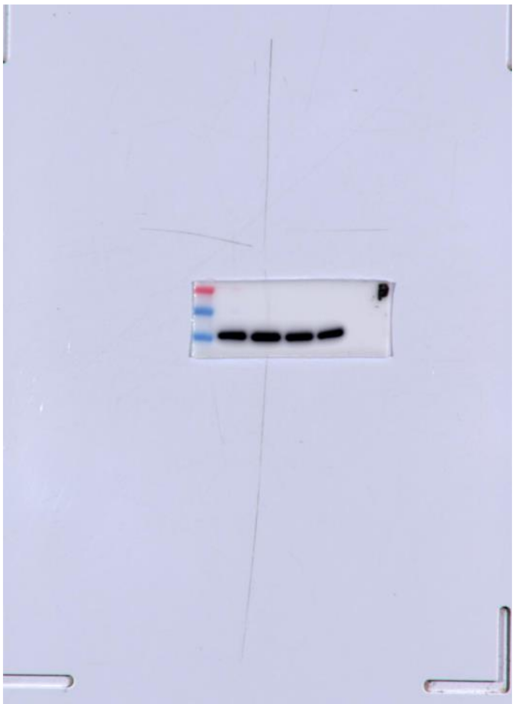

β-Actin

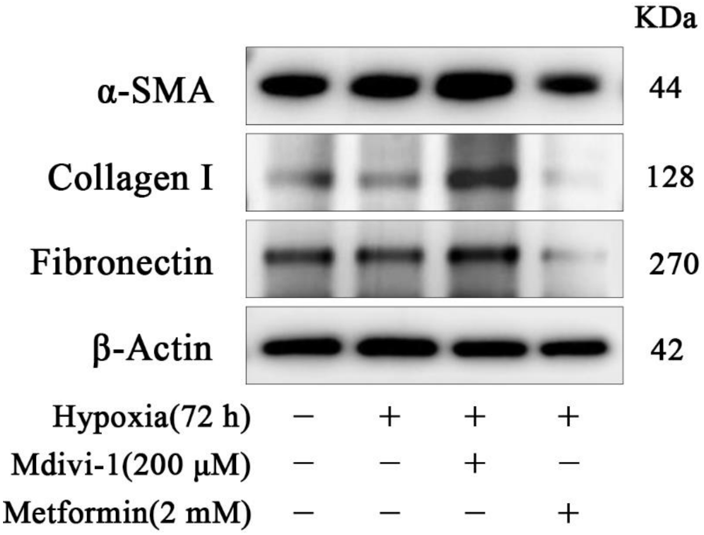

Figure S6. The original WB images of Figure 4G

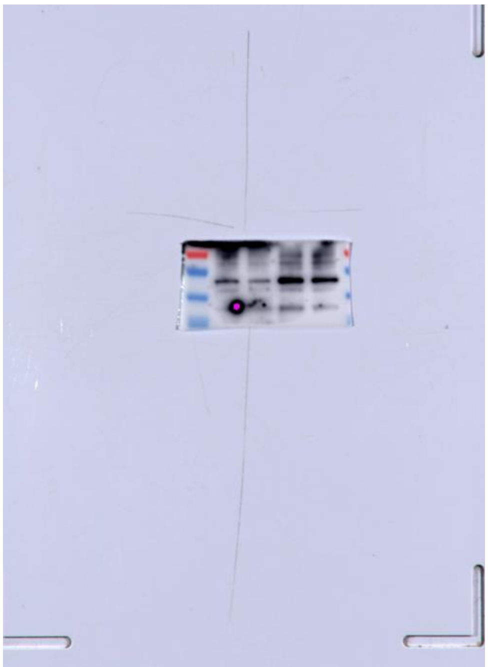

SCL7A11

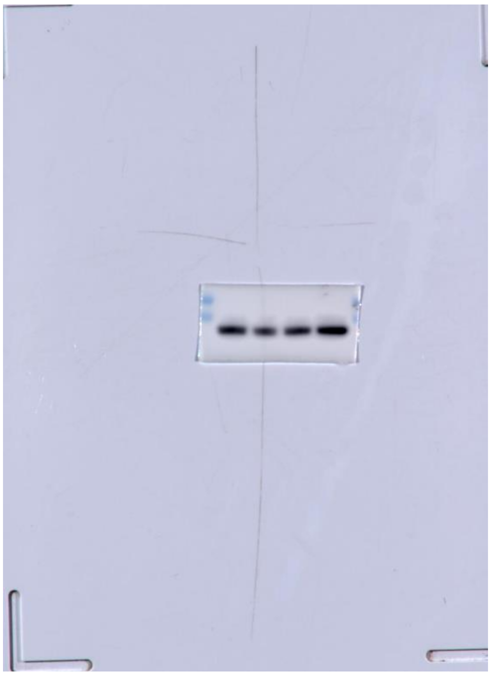

GPX4

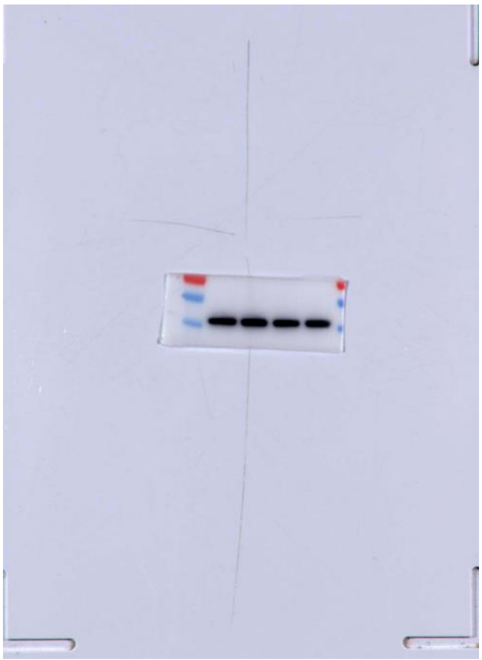

β-Actin

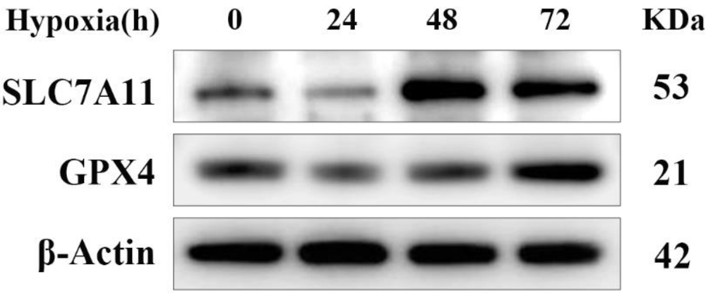

Figure S7. The original WB images of Figure 5A

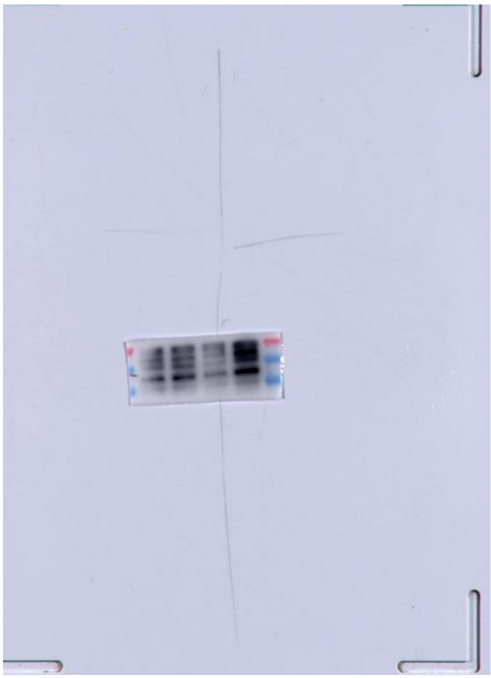

SCL7A11

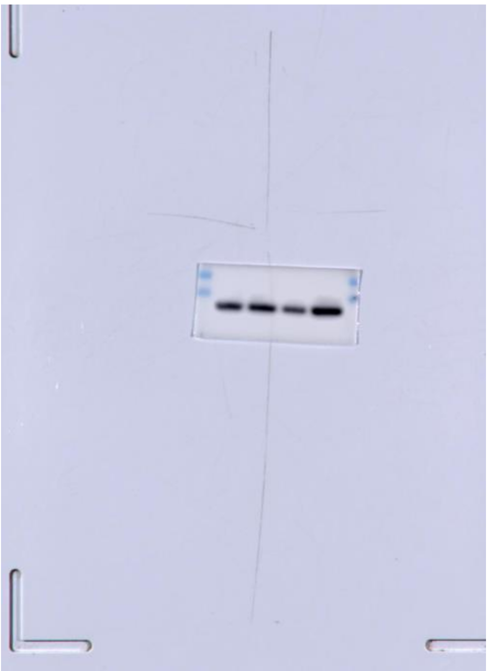

GPX4

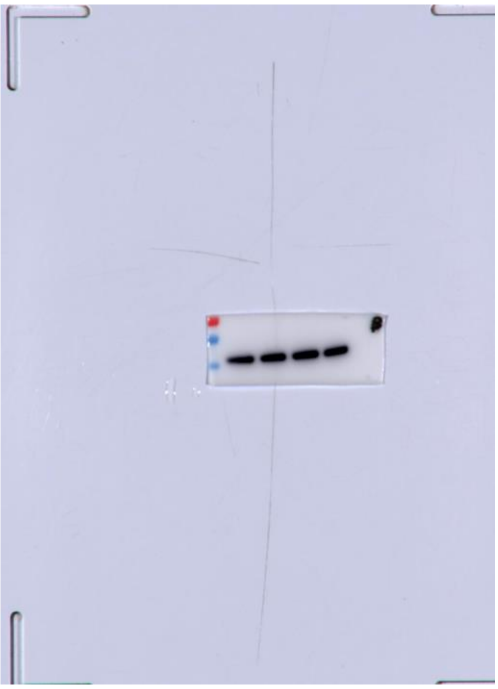

β-Actin

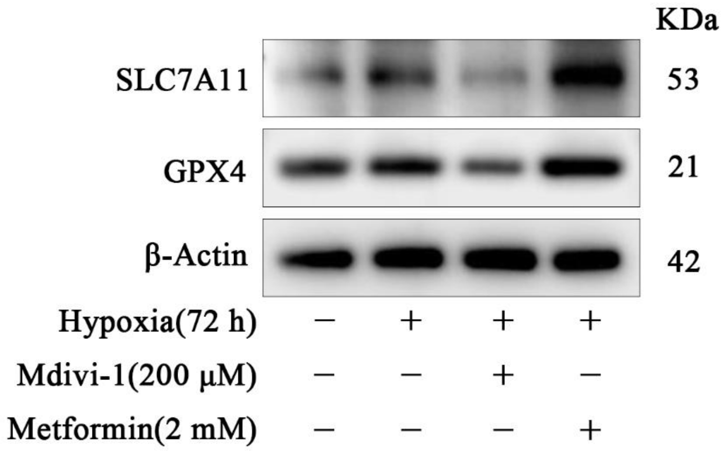

Figure S8. The original WB images of Figure 5E

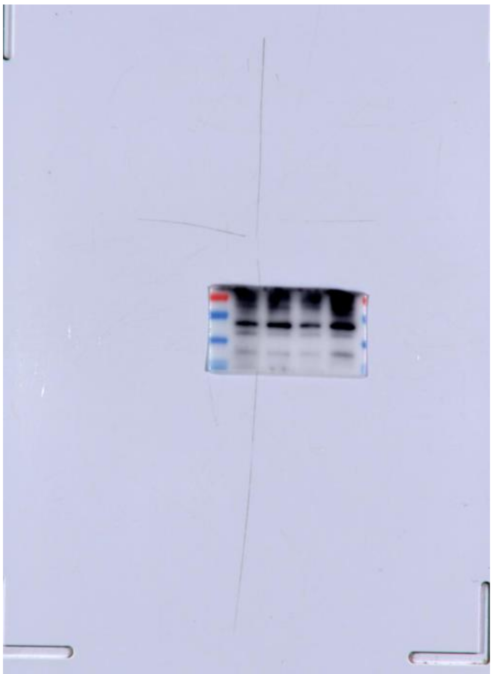

SCL7A11

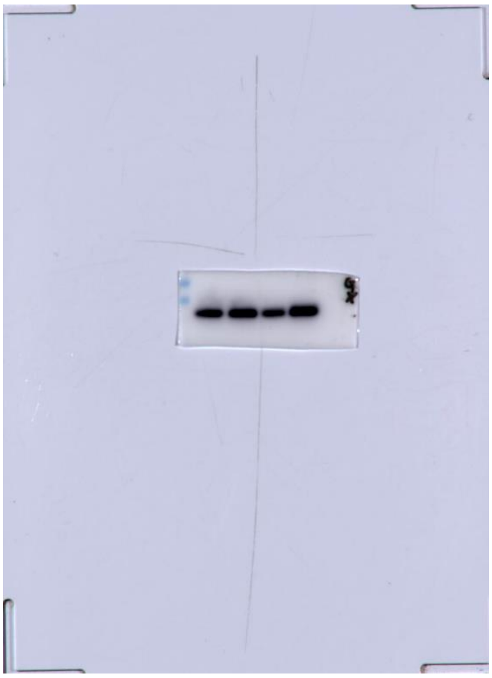

GPX4

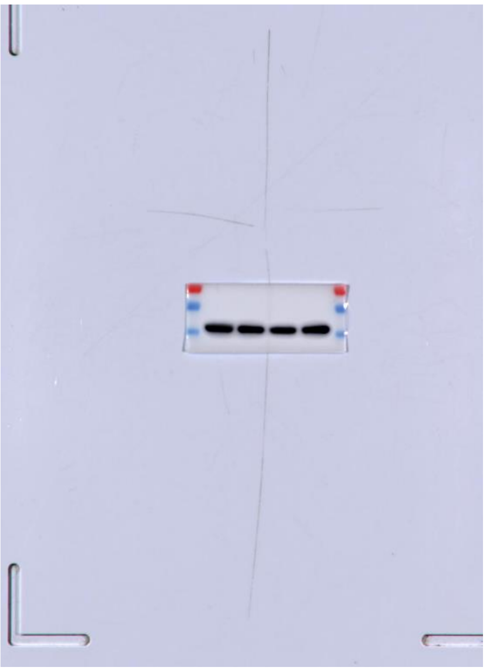

β-Actin

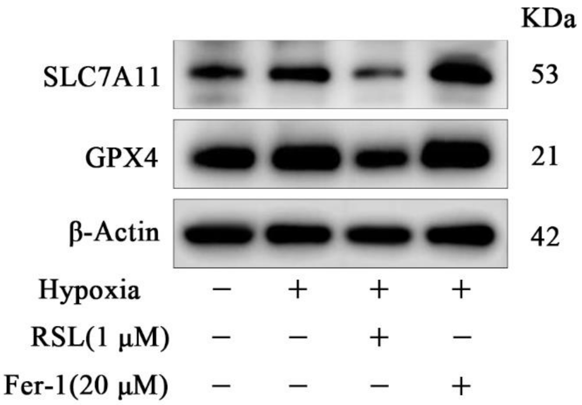

Figure S9. The original WB images of Figure 6C

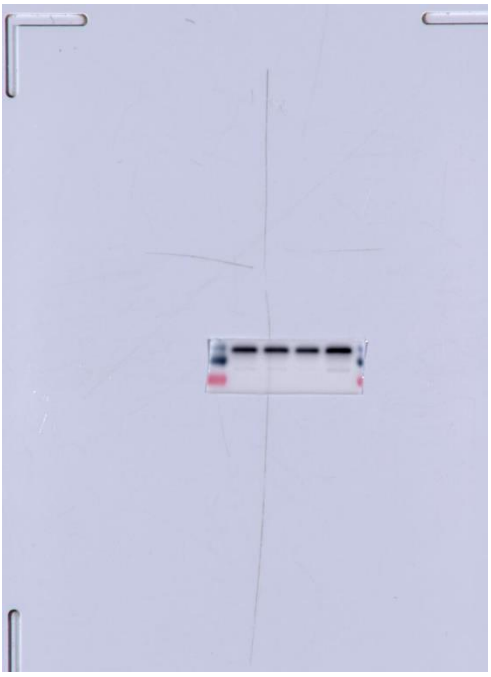

E-cadherin

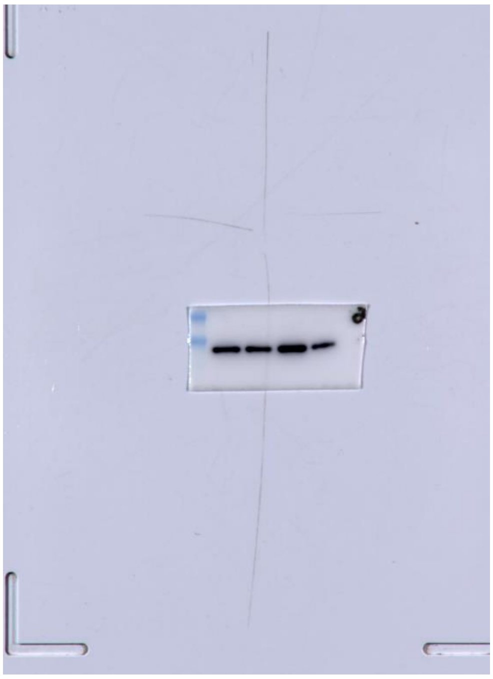

α-SMA

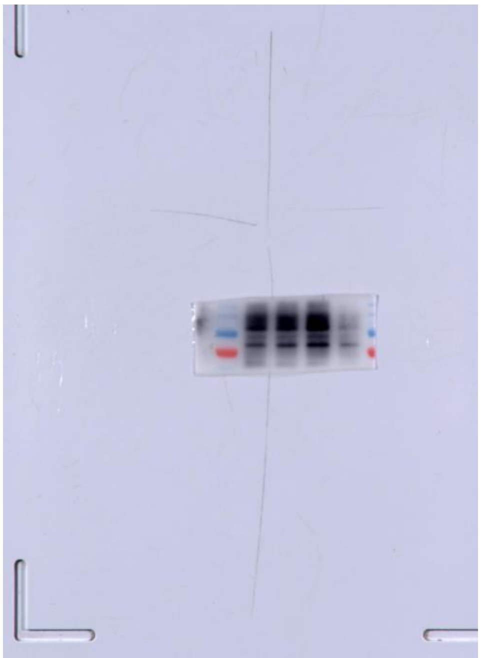

Collagen I

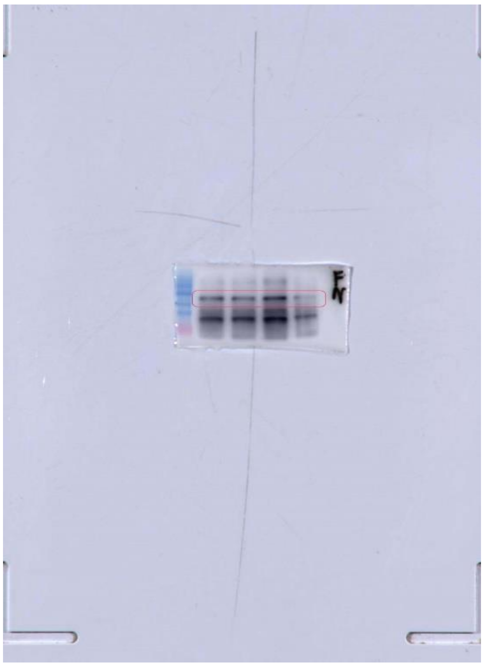

Fibronectin

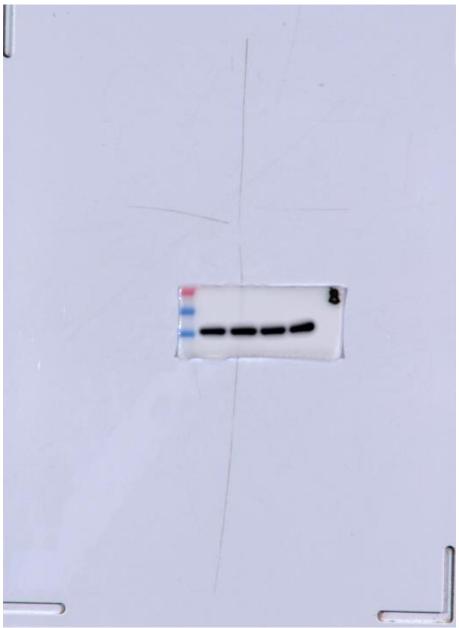

β-Actin

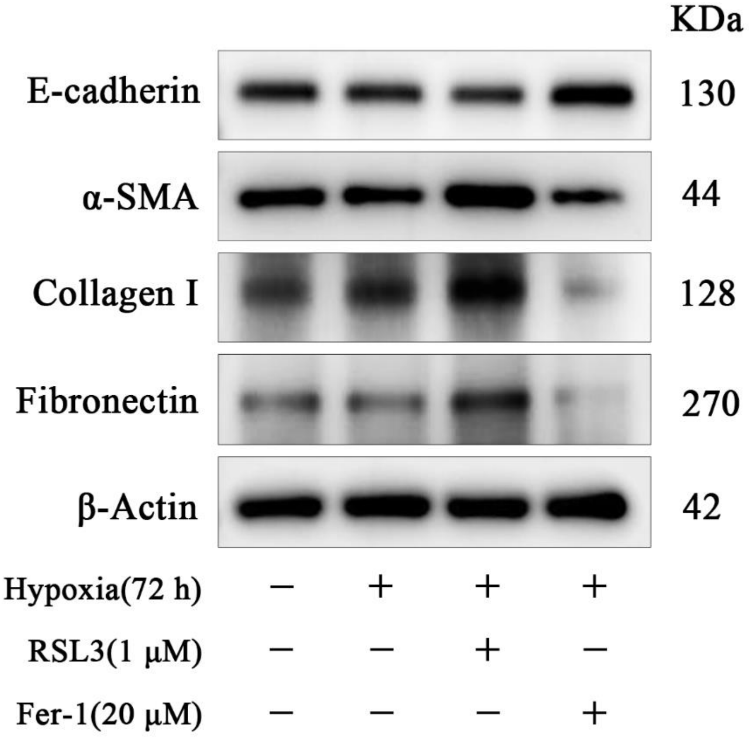

Figure S10. The original WB images of Figure 6H

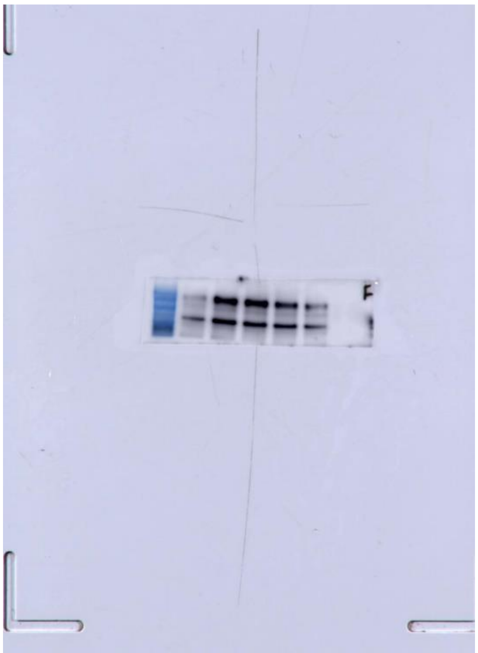

Fibronectin

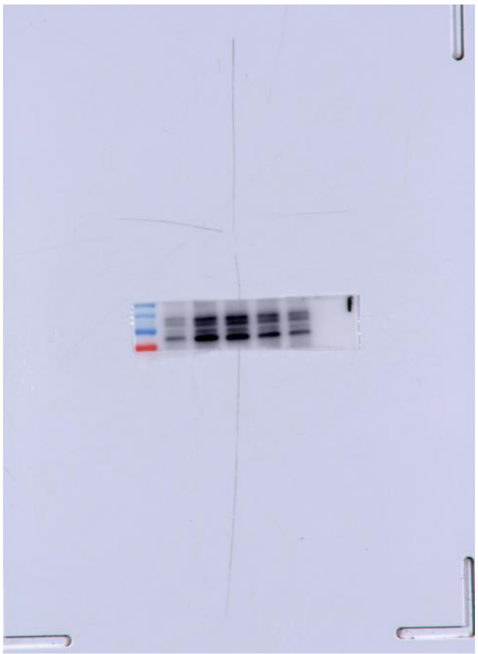

Collagen I

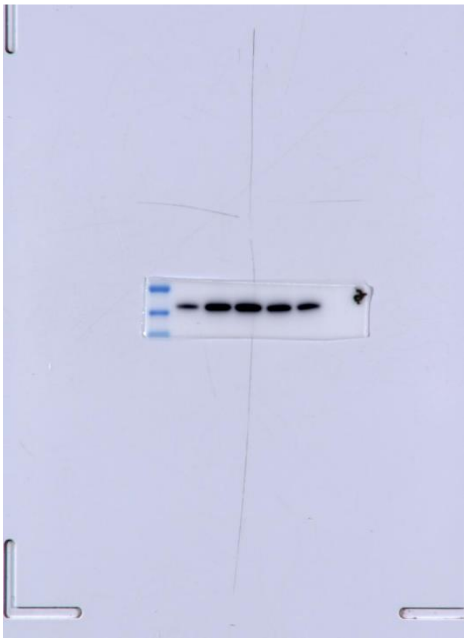

α-SMA

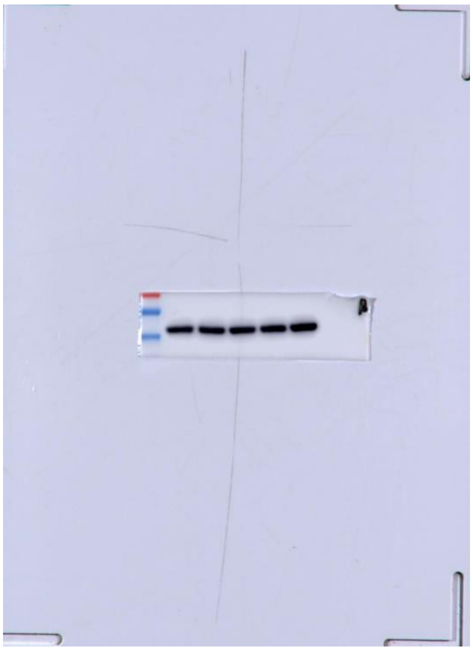

β-Actin

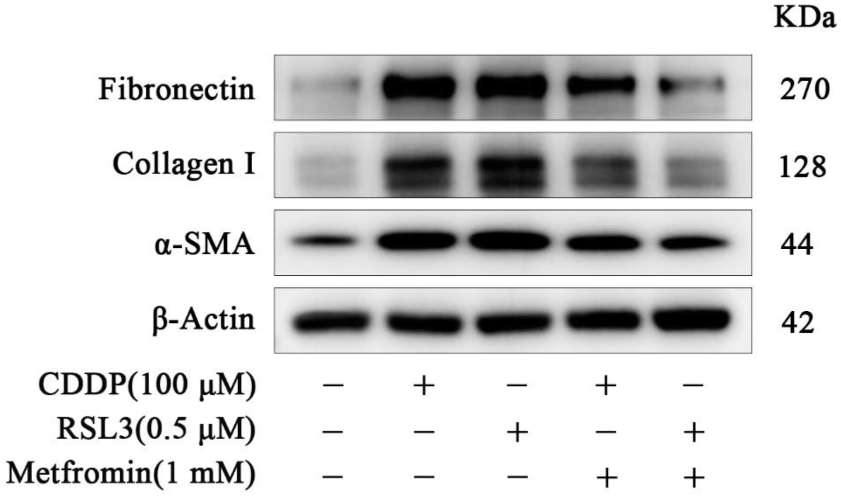

Figure S11. The original WB images of Figure 7G
